# A protocol for computational design of mRNA vaccines with high functionality and specificity

**DOI:** 10.1186/s13062-026-00846-9

**Published:** 2026-06-13

**Authors:** Mennatallah A. Ramadan, Habiba M. ElGohary, Ahmed HI Faraag, Mohamed Mysara

**Affiliations:** 1 Bioinformatics Group, Center for Informatics Science (CIS), School of Information Technology and Computer Science, Nile University, Giza, 12588 Egypt; 2 Medical Biotechnology Department, School of Biotechnology, Badr University in Cairo, Badr City, Cairo, 11829 Egypt

**Keywords:** MRNA vaccine design, Multi-epitope vaccine construct (MEVC), Codon optimization, Translational efficiency optimization, *In silico* cloning, Immune simulation validation, Computational vaccine development

## Abstract

The design of effective mRNA vaccines requires rigorous target selection, optimization, and validation to ensure stability, immunogenicity, specificity, and overall efficacy. A standardized protocol is needed to guide the process from gene selection to in silico optimization of the final vaccine construct. This protocol provides a comprehensive computational framework integrating all stages of mRNA vaccine design by defining the design space, essential criteria, and a step-by-step methodology. To demonstrate its applicability, the protocol is applied to the *SARS-CoV-2* spike protein, targeting both the coding sequence (CDS) and multi-epitope vaccine construct (MEVC). It incorporates immunoinformatics-based epitope selection, population coverage analysis, codon adaptation index optimization, minimum free energy-based RNA secondary structure assessment, target accessibility into a unified computational development workflow. The resulting constructs exhibited strong predicted immunogenicity and broad population coverage, further supported by in silico cloning and immune simulation validation for the optimized MEVC, consistent with prior in vitro and preclinical findings. Collectively, this protocol establishes a standardized computational roadmap for the design of CDS and MEVC mRNA vaccines, facilitating the development of stable, immunogenic, and translationally efficient vaccines against a wide range of pathogens.

**Supplementary Information:**

The online version contains supplementary material available at 10.1186/s13062-026-00846-9.

## Introduction

### Background to mRNA vaccines

**Fig. 1:**
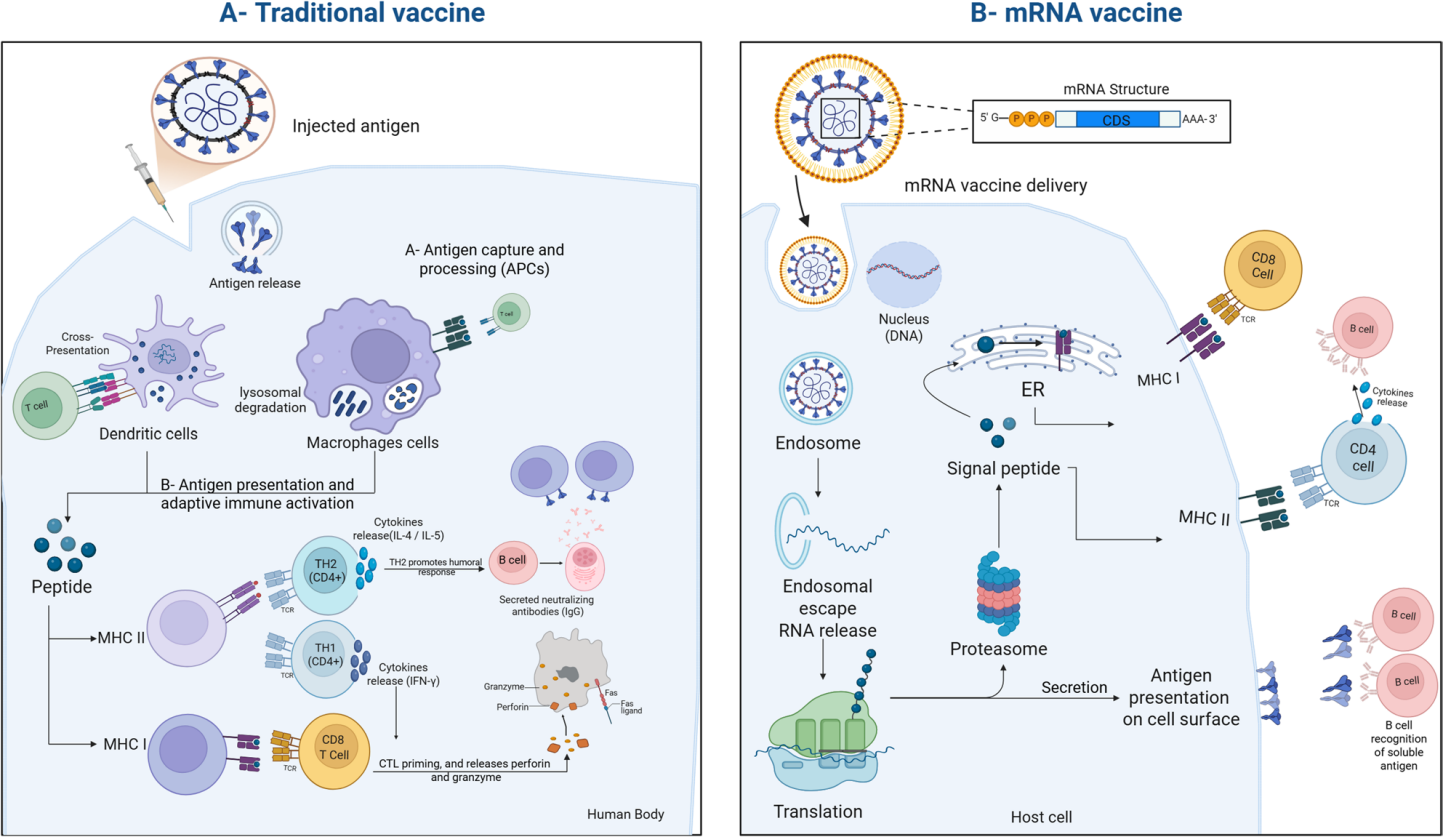
Demonstrating a comparative figure of traditional vaccines and mRNA vaccines. (**A**) traditional vaccines introduce pre-formed antigens derived from inactivated or attenuated pathogens. These antigens are captured by antigen-presenting cells (APCs), including dendritic cells and macrophages, processed intracellularly, and presented as peptides on major histocompatibility complex (MHC) molecules. Peptide–MHC class II heterodimers activate CD4^+^ T helper cells, which differentiate into TH1 and TH2 subsets; TH1 cells promote cytotoxic T-lymphocyte (CTL) activation, whereas TH2 cells support B-cell activation and antibody production. Cross-presentation by dendritic cells enables peptide loading onto MHC class I molecules, leading to CD8^+^ T-cell priming. Activated CTLs mediate effector functions through perforin and granzyme release, while plasma cells derived from activated B cells secrete neutralizing antibodies that circulate systemically in a soluble form. (**B**) mRNA vaccines deliver synthetic mRNA encoding the target antigen into host cells via lipid nanoparticles. Following endocytosis and endosomal escape, the mRNA is translated in the cytoplasm without entering the nucleus. The encoded antigen often contains a signal peptide that directs it to the secretory pathway, allowing both intracellular processing and extracellular release. Intracellularly processed peptides are presented on MHC class I molecules to activate CD8^+^ T cells, whereas secreted or extracellular antigens can be taken up by APCs and presented on MHC class II molecules to activate CD4^+^ T helper cells or directly recognized by B cells. Both pathways induce coordinated cellular and humoral immune responses. Created with BioRender.com

Vaccines have been fundamental in the global fight against infectious diseases, saving millions of lives annually by preventing severe illnesses and outbreaks [1]. They are essential not only for protecting individuals but also for achieving herd immunity, which helps prevent the spread of diseases within communities and protects vulnerable populations [2]. Traditional vaccines (Fig. 1A), such as inactivated or live-attenuated types, involve introducing a weakened or killed version of the pathogen into the body to stimulate an immune response [3]. While effective, these approaches have several limitations, including long production times, the need for high-level biosafety conditions, and potential safety concerns—particularly with live-attenuated vaccines [4]. In addition, the conventional vaccine development process often requires culturing live pathogens in laboratory settings, adding complexity and time to manufacturing, which limits its adaptability to rapidly emerging threats.

mRNA vaccines have emerged as a promising alternative solution to overcome the challenges of traditional vaccines, offering a faster and more flexible approach to vaccine development. Their ability to elicit strong immune responses, coupled with adaptability to different viruses, makes them particularly attractive for combating viral diseases [5]. Unlike traditional vaccines, mRNA vaccines (Fig. 1B) eliminate the need for live pathogens by using synthetic mRNA—a small fragment of genetic material from the virus—that encodes instructions for the host’s cells to produce a specific protein, usually a viral antigen, which triggers an immune response [6]. Once inside the cells, the mRNA is translated into the viral protein by the host’s cellular machinery, which the immune system recognizes as foreign, subsequently prompting antibody production, T-cell activation, and immune memory formation that can quickly respond to future infections if the actual virus enters the body [4]. Consequently, owing to their rapid development and adaptability, mRNA vaccines such as Pfizer-BioNTech’s BNT162b2 and Moderna’s mRNA-1273 were developed against COVID-19 and deployed with remarkable speed during the pandemic [7, 8].

Despite their promise, the design of mRNA vaccine faces several challenges that must be addressed to ensure their success, including mRNA stability, codon optimization, and untranslated regions (UTRs) incorporation [9]. As the field of mRNA vaccine development continues to evolve, existing protocols often tackle these issues in isolation—focusing, for example, mostly on codon optimization or structural stability—without providing a comprehensive and integrated guidelines [10, 11]. Such an approach overlooks critical interdependencies among various design elements, such as the interplay between codon usage, RNA structure, immunogenicity, and UTR selection [12, 13]. A unified protocol that systematically guides researchers from target selection through in silico optimization and validation remains an unmet need in the field [14].

This protocol aims to fill the existing gap by providing a comprehensive computational framework for mRNA vaccine design that integrates key immunoinformatics and structural principles. It defines the design space, establishes essential functional criteria, introduces concepts for optimizing vaccine design, and provides a detailed procedure incorporating codon optimization and structural stability analysis. Using the *SARS-CoV-2* spike protein as a model, the protocol delineates critical design parameters, functional efficiency criteria, and provides a detailed step-by-step procedural guide for in silico vaccine sequence development. By unifying all key elements into a cohesive pipeline, it standardizes the design process and supports the development of stable, immunogenic, and translationally efficient mRNA vaccines across diverse pathogens.

### Defining design space for mRNA vaccine efficiency

#### Design space for mRNA vaccine

Optimizing the design space is crucial for developing mRNA vaccines that encompass several key components including UTRs, coding sequence (CDS), mRNA secondary structure, sequence length and GC content. The 5‘UTR and 3’ UTR are essential in regulating translation efficiency, stability, and overall mRNA expression, with the 5‘UTR specifically contributing to ribosome recruitment and binding and 3’ UTR influencing mRNA stability and intracellular localization [15]. At the 5′ end, the mRNA includes a 5′ cap, which facilitates ribosome recognition, translation initiation, and protection from exonuclease degradation [16]. Following the 5‘cap is the 5’ UTR, within which the Kozak sequence is embedded—a conserved nucleotide motif surrounding the start codon, that guides the ribosome to the correct initiation site [17].

Following the 5’ UTR, the CDS encodes a protein that often contains structural domains with distinct functions governing protein folding, localization, and biological activity. Within these domains, conserved short patterns may occur that contribute to specific functional roles and are commonly referred to as motifs. In the context of vaccine design, particular attention is given to antigenic regions of proteins that may contain immunogenic epitopes. These immunogenic epitopes are often found within specific functional domains of the protein, particularly within surface-exposed or extracellular regions that are accessible to immune surveillance and represents the primary targets of vaccine-induced immune responses [5, 18]. Following translation, intracellularly expressed antigens are processed through proteasomal or endosomal pathways, generating peptide epitopes that are presented on the cell surface in association with MHC class I or class II molecules for T-cell recognition, while intact or secreted antigens may be directly recognized by B cells [4]. Toward the end of the CDS, the downstream 3′ UTR plays an important role in regulating mRNA stability, localization, recycling, and translation termination [19]. This region interacts with cellular machinery to protect the mRNA from degradation, thereby extending its half-life and supporting sustained protein production. Proper optimization of the 3′ UTR involves the incorporation of regulatory elements that enhance translation efficiency and prolong mRNA stability, with specific sequence features enabling productive interactions with protective cellular factors [20].

#### Secondary structure, length and GC content of mRNA vaccine

The secondary structure of mRNA is another key design element, as its folding can directly influence translation efficiency. Features such as hairpins and loops, particularly near the 5′ UTR or start codon, can impede translation efficiency [21]. UTRs are not merely linear sequences but often form these complex secondary structures that can create barriers to ribosome scanning and translation initiation, especially in the 5′ UTR [15]. Other elements in the design space are the length and nucleotide composition of the mRNA sequence that significantly impact mRNA stability and translation efficiency. Longer mRNA sequences are generally more prone to degradation and often require stabilizing modifications and length optimization [22]. Additionally, the GC content, or the proportion of guanine and cytosine nucleotides, affects the mRNA’s stability and secondary structure [23]. High GC content may form stable secondary structures that hinder translation, while low GC content can result in less stable mRNA [24]. Thus, efficient mRNA design must holistically consider 5′ and 3′ UTRs, CDS, secondary structure, sequence length, and GC content to ensure stability, strong protein expression, and minimal immunogenicity.

### Essential criteria for mRNA vaccine functionality

The successful design of mRNA vaccine sequences hinges on critical criteria that ensure their functionality within the host, including the 5′ and 3′ UTRs, CDS, secondary structure stability, and immunogenicity. These factors determine how effectively the mRNA produces the target antigen while maintaining stability and safety. Optimizing the 5’ UTR selection must be carefully done to promote effective ribosome binding and avoid forming highly stable secondary structures that could hinder translation initiation or may prevent translation [4]. Selecting UTRs from host sources known to enhance mRNA stability and translation ensures compatibility with the host’s translational machinery, thereby promoting efficient protein synthesis [25]. Other criteria for UTR selection include length and structural complexity, with shorter 5’ UTRs generally preferred, as longer, highly structured regions may reduce translation efficiency [15]. Upstream start codons (AUGs) or upstream open reading frames (uORFs) within regulatory regions, especially in the 5′ UTR, should be avoided as they interfere with translation of the main open reading frame (ORF) through premature initiation or ribosome scanning disruption [26, 27]. Codon usage can also influence translation efficiency—mainly within the CDS—by modulating elongation rates, as will be further illustrated in this section. The combination of the MFE of both the 5′ UTR and the CDS should be calculated to assess overall stability without hindering translation, while still protecting the mRNA from degradation [28]. MFE should be evaluated comprehensively since UTR–CDS interactions affect both stability and translation. UTR optimization further requires balancing this structural stability, avoiding excessive folding that impairs translation while maintaining adequate protection [29, 30]. The 5′ UTR of human *TMSB10 or Ces1d*, for example, have been shown to enhance antigen-specific humoral and cellular immune responses when used in mRNA vaccines encoding the receptor-binding domain (RBD) of *SARS-CoV-2*, demonstrating superior expression in dendritic cells [31]. Likewise, the 5′ UTR of human β-globin is well-documented for its strong ability to promote cap-dependent translation initiation, thereby boosting protein synthesis in mRNA applications [32].

The 3′ UTR also requires careful consideration to avoid unintended immune responses and prolong its half-life, as it is vital for mRNA stability and cellular localization. Selecting 3′ UTR sequences that promote stability while avoiding immune-triggering motifs or premature degradation is critical. For instance, incorporating polyadenylation signals within the 3’ UTR can extend mRNA half-life and ensure sufficient protein production [33, 34]. The 3′ UTRs of human *Apo A-II* and *AP3B1* have proven effective in extending mRNA half-life and supporting high protein output in vivo, especially when paired with strong 5′ UTRs [31]. In comparative screening studies assessing multiple UTR combinations, the pairing of the 5′ UTR from *TMSB10* with the 3′ UTR *of Apo A-II* emerged as one of the most effective human UTR configurations when combined with viral coding sequences such as that of the *SARS-CoV-2* spike protein, yielding significantly increased antigen expression and immunogenicity. These findings support the rationale for utilizing empirically validated UTRs in combination with viral CDS to optimize mRNA vaccine performance.

The CDS within mRNA contains crucial elements that significantly impact vaccine effectiveness. For instance, short motifs of 6–15 amino acids are often associated with receptor binding or immune activity, whereas longer motifs as 20–50 amino acids may correspond to structural elements such as fusion peptides or heptad repeats [35]. Additionally, certain regions of the encoded proteins, particularly those that are surface-exposed, may harbor the antigenic epitopes recognized by the immune system [36]. Epitopes are generally classified as linear and conformational peptide types. Linear epitopes are often favored, as they consist of continuous amino acid sequences meaning the residues are adjacent in the primary sequence, making them easier to identify and more reliably predicted for peptide-based vaccine design [18]. In contrast, conformational epitopes are discontinuous, formed by amino acids that are distant in sequence but brought together in the folded protein structure. Effective epitope selection should include both CD8^+^ and CD4^+^ T-cell epitopes, requiring compatibility with MHC class I and class II human leukocyte antigen (HLA) molecules to ensure a coordinated cellular immune response [37, 38]. HLA molecules are cell-surface proteins responsible for presenting peptide antigens to T cells, thereby enabling immune recognition and activation. The binding affinity between the epitope and HLA determines how efficiently the antigen is recognized and triggers immune response. Signal peptides optimization is another essential aspect for directing newly synthesized proteins into the secretory pathway, influencing protein folding, secretion, and overall expression levels [39]. These short, N-terminal sequences are found in both prokaryotes and eukaryotes and play a vital role in recombinant protein production [39, 40].

The immunogenicity must also be optimized to trigger an appropriate immune response without causing adverse effects such as autoimmunity [41]. The innate immune system recognizes exogenous mRNA via pattern recognition receptors, including endosomal Toll-like receptors (TLRs) and cytosolic RNA sensors such as RIG-I and MDA5, which can lead to inflammation or transcript degradation if excessively activated [42]. Overactivation of these pathways may amplify type I interferon signaling, promote local inflammatory responses and ultimately reduce antigen expression [43]. Consequently, candidate antigen sequences should be systematically screened for antigenicity, allergenicity, and toxicity as essential safety filters during vaccine design [44]. Population coverage analysis also represents a critical design criterion in epitope-based vaccines. It refers to the proportion of individuals within a given population who are predicted to mount an immune response based on HLA allele distribution. Because HLA polymorphisms vary across ethnicities and geographic regions, selecting epitopes that bind to multiple common HLA alleles increases the likelihood of broad vaccine effectiveness at the population level [45].

To enhance immunogenic breadth and ensure the induction of both cellular (T-cell-mediated) and humoral (antibody-mediated) immune responses, multiple immunodominant epitopes are integrated into a multi-epitope construct for MEVC formation, allowing simultaneous presentation of cytotoxic T lymphocytes (CTL), helper T lymphocytes (HTL) and B-cell epitopes within a single sequence [46]. The inclusion of an adjuvant is essential, as short peptide epitopes alone often exhibit limited intrinsic immunogenicity; therefore, molecular adjuvants are incorporated to enhance immune activation. Among these, β-defensin is commonly used due to its ability to stimulate innate immunity, promote dendritic cell maturation, enhance T-cell responses and its use has been consistently reported in multi-epitope vaccine studies [47–49]. Essential aspect here is the rational linker selection which is critical to prevent the formation of junctional epitopes and to facilitate correct epitope presentation. Once the multi-epitope sequence is assembled, it should be evaluated for immunogenicity and for physicochemical properties, including molecular weight, stability, hydrophobicity (GRAVY, the average hydropathy score, where positive values indicate hydrophobicity and negative values indicate hydrophilicity), secondary structure tendencies, and predicted in vivo and in vitro half-life [50].

Another critical aspect of mRNA design is codon optimization which is fundamental in the design space. Codons determine amino acid incorporation during protein synthesis. Organisms exhibit species-specific codon usage preferences, referred to as codon bias, whereby certain codons are more efficiently recognized by the translational machinery, resulting in enhanced protein expression [51]. Optimization of codon usage bias should be aligned with host preferences as human codon usage to enhance translation efficiency and reduce ribosomal stalling [52]. Chemical nucleotide modification is an important factor in mRNA functionality that complements sequence-level optimization in modern mRNA vaccine design [53]. In current platforms, standard ribonucleotides are often replaced with natural or synthetic analogues to enhance stability, translational efficiency, and tolerability. Uridine is commonly substituted with N1-methylpseudouridine (m^1^ Ψ) or pseudouridine (Ψ), as used in clinically approved *SARS-CoV-2* mRNA vaccines, reducing innate immune activation while enhancing protein expression in vivo [53]. Additional modifications, including 5-methylcytidine (m5C), N6-methyladenosine (m6A), and 2-thiouridine (s2U), have also been explored for their roles in improving mRNA stability, translation, and immunogenicity. Comparative studies show that Ψ- and m^1^ Ψ-modified mRNA increase duplex stability and outperform unmodified constructs in protein production while maintaining improved safety profiles [54–56].

The secondary structure is another criterion that must be carefully considered as naturally folded mRNA can hinder ribosome access or promote degradation. Predicting folding patterns of MEVC/CDS vaccine and identifying stable regions allows adjustments to minimize unwanted folding [57]. Structural stability is commonly assessed by minimum free energy (MFE), a thermodynamic measure of RNA folding; lower MFE values indicate more stable structures that can protect mRNA from degradation and promote efficient translation while high MFE increase degradation risk [11, 58]. As MFE scales with RNA length and complexity, values must be interpreted relative to length and function. The length and sequence composition of UTRs and CDS also influence secondary structure formation, which must be minimized to facilitate ribosome binding [15]. Optimization of UTRs and the 5’ cap structure helps reduce unintended innate immune sensing and improves transcript stability [59, 60]. These steps should be performed alongside assessment of target accessibility to ensure mRNA availability to the translational machinery and additional two-dimensional (2D) structure analysis for the optimized MEVC to predict α-helices, β-sheets, and coil regions within the protein sequence of the vaccine construct [58].

Cloning efficiency is another important consideration, as suboptimal insertion can compromise construct amplification and necessitate further optimization. Accordingly, the MEVC or CDS constructs are introduced into a suitable expression system, most commonly an *Escherichia coli* plasmid such as pET-28a(+), to evaluate protein production efficiency [61]. In this context, the BamHI restriction enzyme recognition site is typically incorporated at the N-terminal 5′ end of the insert, while the XhoI site is introduced at the C-terminal 3′ end, ensuring correct orientation of the construct relative to the promoter and minimizing the likelihood of vector self-ligation [62–64].

Once the final construct is obtained, several aspects should guide the evaluation of vaccine-induced immune responses. Immune simulation serves as an essential validation step, where the immunization strategy—particularly dose, route, and scheduling, including prime–boost regimens—shapes the response [63]. Both humoral and cellular immunity should be assessed, including antibody production, neutralization capacity, and T-cell activation and phenotype. Cytokine and interleukin profiles provide insight into immune signaling and the balance between effective and excessive inflammation. The development of immunological memory through B- and T-cell responses should also be evaluated, together with antigen clearance to determine immune efficiency. Safety and tolerability remain critical to ensure a well-regulated response. The mRNA delivery system, most commonly lipid nanoparticles, further supports vaccine performance by protecting mRNA, promoting cellular uptake, and enabling endosomal escape for efficient translation [4, 9, 65]. Advances in lipid nanoparticle composition and formulation have demonstrated that delivery efficiency strongly influences antigen expression levels, immune activation, and overall vaccine performance [66, 67]. Although delivery optimization is beyond the scope of this protocol, its impact on vaccine performance is acknowledged as a critical factor for achieving effective and safe antigen expression in vivo.

### Design mRNA vaccine for specificity

**Fig. 2:**
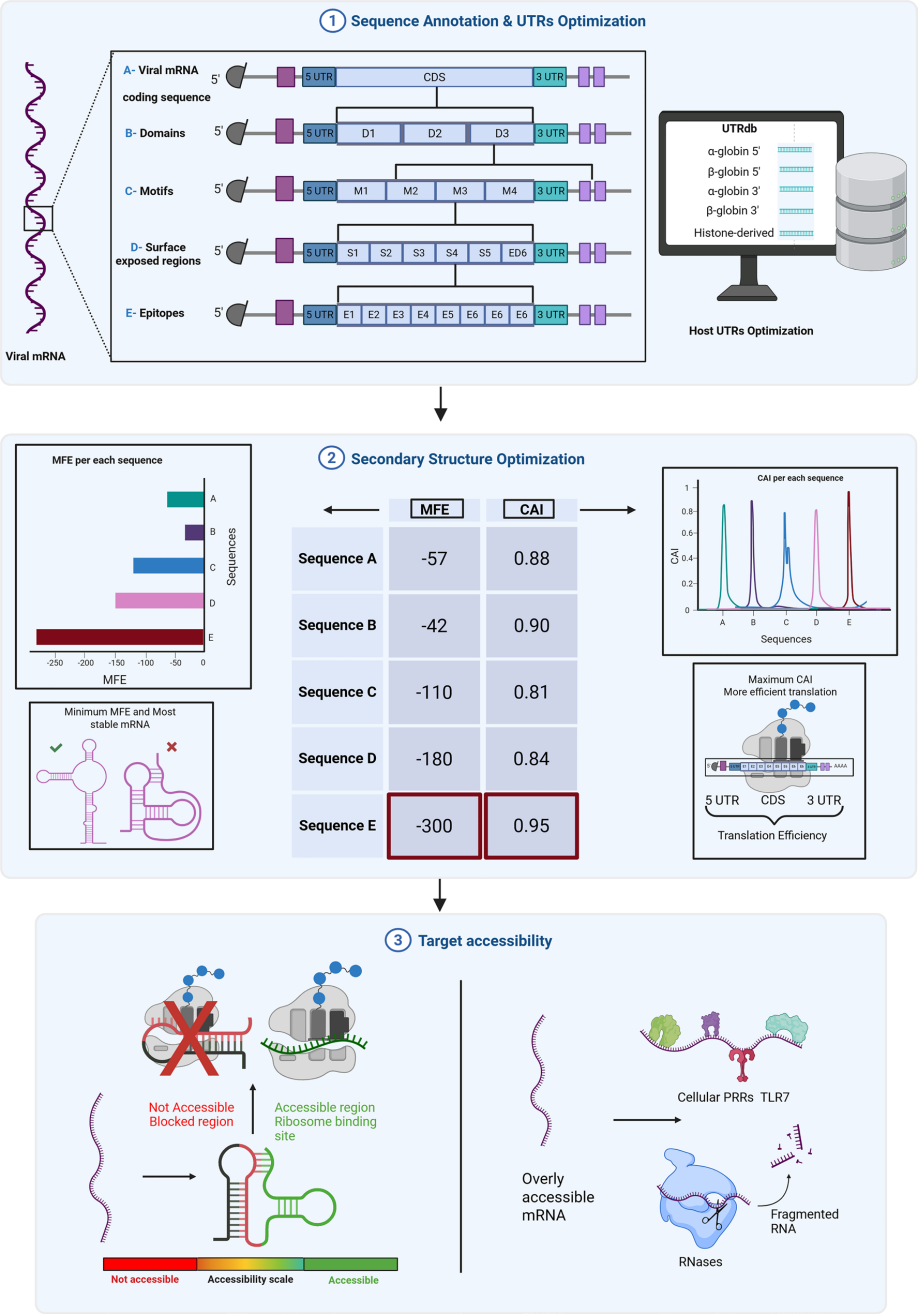
Optimization of mRNA vaccine design. The crucial three steps in mRNA vaccine sequence optimization are presented in the figure: (1) sequence annotation & UTR optimization, where viral mRNA coding sequence is annotated into domains, motifs, surface-exposed regions, and epitopes. The 5′ and 3′ UTRs are optimized for stability and translation efficiency. (2) secondary structure optimization, and stability of different sequences calculated using MFE, a lower MFE indicating higher stability. Sequence E, MFE (−300), most stable. Codon adaptation index (CAI) is also computed, and sequence E has the maximum CAI (0.95), indicating greater translation efficiency. (3) target accessibility, where mRNA is optimized for ribosomal binding and circumvented for undesirable interactions. Accessible mRNA is too accessible and can engage immune sensors like TLR7 or lead to RNase-mediated degradation and compromise vaccine efficacy. Created with BioRender.com

In mRNA vaccine design, two main strategies are widely adopted: the full-length spike protein approach and the multi-epitope vaccine construct (MEVC) approach. The full-length strategy, as demonstrated in vaccines like Pfizer-BioNTech’s BNT162b2 and Moderna’s mRNA-1273, encodes the entire *SARS-CoV-2* spike protein to induce a broad immune response thus stimulating both B and T cells, offering strong protection. Conversely, the multi-epitope approach targets highly conserved immunodominant regions such as the receptor-binding domain (RBD), improving specificity and reducing potential adverse effects [68, 69]. While the full-length design offers broader immunogenicity, the multiepitope approach adapts faster to viral mutations and may lower the risk of unwanted immune responses [44]. The optimization of mRNA vaccine design involves several steps accounting for whether the target region within the virus is known or yet to be defined (Fig. 2). If the target protein is not identified, the entire viral genome should be screened to identify the potential vaccine targets then the target protein sequences should be aligned to determine the conserved regions within these sequences to ensure effectiveness across multiple strains. The screening is done through in-depth annotation of the protein sequences to identify essential structural and functional components within the protein and their immunogenicity, such as domains, extracellular domains of transmembrane proteins or surface-exposed regions, motifs, and epitopes.

Epitope identification is subsequently performed using computational tools, which can be categorized into cytotoxic T lymphocyte (CTL; MHC class I-restricted), helper T lymphocyte (HTL; MHC class II-restricted), linear B-cell, and conformational B-cell epitope prediction [70]. For T cells, Binding affinity is evaluated using IC₅₀ values, which represent the half-maximal inhibitory concentration and reflect peptide–MHC binding strength; IC₅₀ thus serves as a key immunological criterion for prioritizing protein regions most likely to be presented by prevalent HLA molecules, guiding their inclusion in vaccine constructs [71]. Lower IC₅₀ values indicate stronger binding and higher likelihood of immune recognition; epitopes with IC₅₀ <50 nM are classified very strong binders, while those < 500 nM are considered regular binders [72]. Epitopes are further prioritized based on prediction-derived metrics, particularly the percentile rank and prediction score. Lower percentile ranks correspond to higher binding affinity, with values below 1% considered strong binders and values up to ~1.5–2% regarded as moderate- to high-affinity binders, while higher prediction scores indicate stronger peptide–MHC interactions [73]. For B-cell epitope prediction, higher scores are preferred, as they indicate a greater likelihood that the region represents a B-cell epitope and is recognized by antibodies. In vaccine design, epitope selection and filtering are based on a combination of percentile rank, high score and the frequency of experimental validation, such as the number of supporting assays reported in Immune Epitope Database (IEDB) [74]. Each assay represents an independent experimental validation performed ex vivo, in vitro, or in vivo and is linked to a reference identifier corresponding to the original study. Epitopes supported byhigh number of experimental assays are considered more reliable and prioritized for robust vaccine design [75]. The target’s specificity and immunogenicity are then validated by confirming that the selected epitopes elicit strong immune response, avoiding unintended immune interactions and allergenicity. Once the non-allergenic, non-toxic and antigenic epitopes are identified, their coding selection and structural stability are also evaluated by assessing translation efficiency, folding stability, and preservation of the original amino acid content. Following this, population coverage analysis is performed in epitope-based vaccine design, as it evaluates whether the selected T-cell epitopes MHC class I and MHC II and their associated strong binding HLA alleles can provide broad immune coverage across different ethnicities and geographical regions worldwide.

**Fig. 3:**
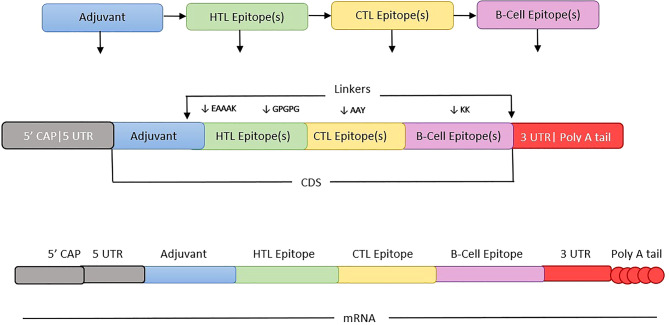
Schematic representation of the multi-epitope mRNA vaccine construct. The design includes an N-terminal adjuvant followed by HTL, CTL, and B-cell epitopes linked using EAAAK, GPGPG, AAY, and KK linkers to facilitate proper processing and presentation. The coding sequence is flanked by regulatory elements, including the 5′ cap and 5′ UTR upstream and the 3′ UTR with a poly (A) tail downstream, ensuring efficient translation and stability. Created with drawio.com and MS word

The shortlisted epitopes can be assembled into a single chimeric coding sequence of MEVC. The construct design follows a rational and ordered arrangement in which the immunostimulatory adjuvant sequence is first incorporated at the N-terminus to enhance immune activation of the construct, optionally preceded by a signal peptide if secretion is desired. Typically, following the N-terminal adjuvant, becomes the core of the construct, where HTL epitopes are positioned first to initiate and support immune activation through CD4^+^ T-cell responses, followed by CTL epitopes to promote efficient MHC class I presentation and cytotoxic T-cell activation, and finally B-cell epitopes to enhance antibody-mediated responses and antigen recognition. These epitopes are connected using functionally validated short peptide linkers to preserve their independent immunological properties [46]. The AAY linker is commonly used between CTL epitopes to enhance proteasomal cleavage and MHC class I presentation, the GPGPG linker is typically used between HTL epitopes to promote antigen processing and structural flexibility, and the KK linker is used to improve epitope separation and lysosomal processing (Fig. 3). Following assembly, the complete multi-epitope construct is evaluated in silico as a unified sequence to assess antigenicity, allergenicity, toxicity, and physicochemical properties for minimizing the risk of unfavorable biological responses during downstream development [76, 77].

After pinpointing the immunologically relevant regions, the CDS corresponding to the selected protein or MEVC is retrieved. If starting from a consensus protein sequence, the equivalent CDS is derived through reverse translation. This CDS is then optimized in the next step through humanization and codon optimization to improve translation efficiency, aligning with host as human codon usage preferences which is normally assessed by a codon adaptation index (CAI) value. The next step in the design involves an iterative refinement process using the *LinearDesign* algorithm [11]. The *LinearDesign* algorithm focuses on optimizing the CAI and the MFE of the CDS region of the selected protein or MEVC [78]. CAI values near 1.0 indicating strong optimization and typically correlating with higher translation efficiency and protein expression, while GC content should typically fall within the range of 30–70% to ensure transcriptional stability [8, 78]. Following the optimization of the coding sequence based on CAI, calculate CAI and MFE values for the MEVC in addition to the full CDS of the spike protein. When using algorithms or tools that perform codon optimization without incorporating MFE calculations, then mRNA secondary structure and accessibility should be assessed after coding optimization. Additional 2D structure analysis is recommended for the optimized MEVC using tools based on position-specific scoring matrices to predict α-helices, β-sheets, and coil regions, while assigning confidence scores to each residue, where higher scores indicate greater prediction reliability [79].

Computational approaches, such as in silico screening, identify optimal signal peptides, ensuring the vaccine protein is efficiently expressed and presented to the immune system [80, 81]. Subsequently, in silico cloning is performed as a validation step to assess the feasibility of construct integration into an expression system and to ensure the success of subsequent experimental validation. In this context, the designed sequence, referred to as the insert, is virtually integrated into an expression vector, typically a plasmid. The pET-28a(+) vector is commonly used due to its strong T7 promoter, efficient transcriptional control, and compatibility with *E. coli* expression strains [63, 64]. During in silico cloning, restriction enzymes such as BamHI and XhoI are selected to ensure correct orientation and prevent self-ligation, as they are present in the multiple cloning site (MCS) and generate non-compatible cohesive ends for directional cloning. Specifically, the BamHI recognition site is introduced at the N-terminal (5′) end of the insert, while the XhoI site is incorporated at the C-terminal (3′) end, ensuring proper orientation of the construct within the vector. Following in silico cloning, immune simulation serves as the last in silico validation step that provides a preliminary assessment of the vaccine’s ability to induce an immune response prior to experimental testing [63]. A key consideration in this process is the simulation of a realistic vaccination regimen, typically following a single-dose or multi-dose (prime–boost) schedule, where repeated antigen exposures are used to mimic initial immunization and subsequent booster doses. This includes assessment of antibody production, T-cell activation, cytokine signaling, memory formation, and antigen clearance under simulated immunization conditions [82]. A progressive reduction in antigen levels after each simulated dose is generally indicative of an effective immune response.

The next optional step is UTR optimization, integrating 5′ and 3′ UTRs with the CDS/MEVC to balance structural stability and translation efficiency. In this optional step, Host-derived UTRs as human UTRs are combined with viral CDS, and multiple UTR combinations are systematically tested, with total combinations calculated as (number of 5′ UTRs × number of 3′ UTRs). As for mRNA vaccine applications (Fig. 3), the optimized coding sequence or MEVC is incorporated into a complete mRNA architecture that includes a 5′ cap structure to initiate translation, a 5′ untranslated region to enhance ribosome binding, the coding sequence encoding the multi-epitope construct, a 3′ untranslated region to improve transcript stability, and a poly(A) tail to further enhance stability and translation efficiency [83, 84].

#### Procedure: applying the protocol to the spike protein of *SARS-CoV-2*

**Fig. 4:**
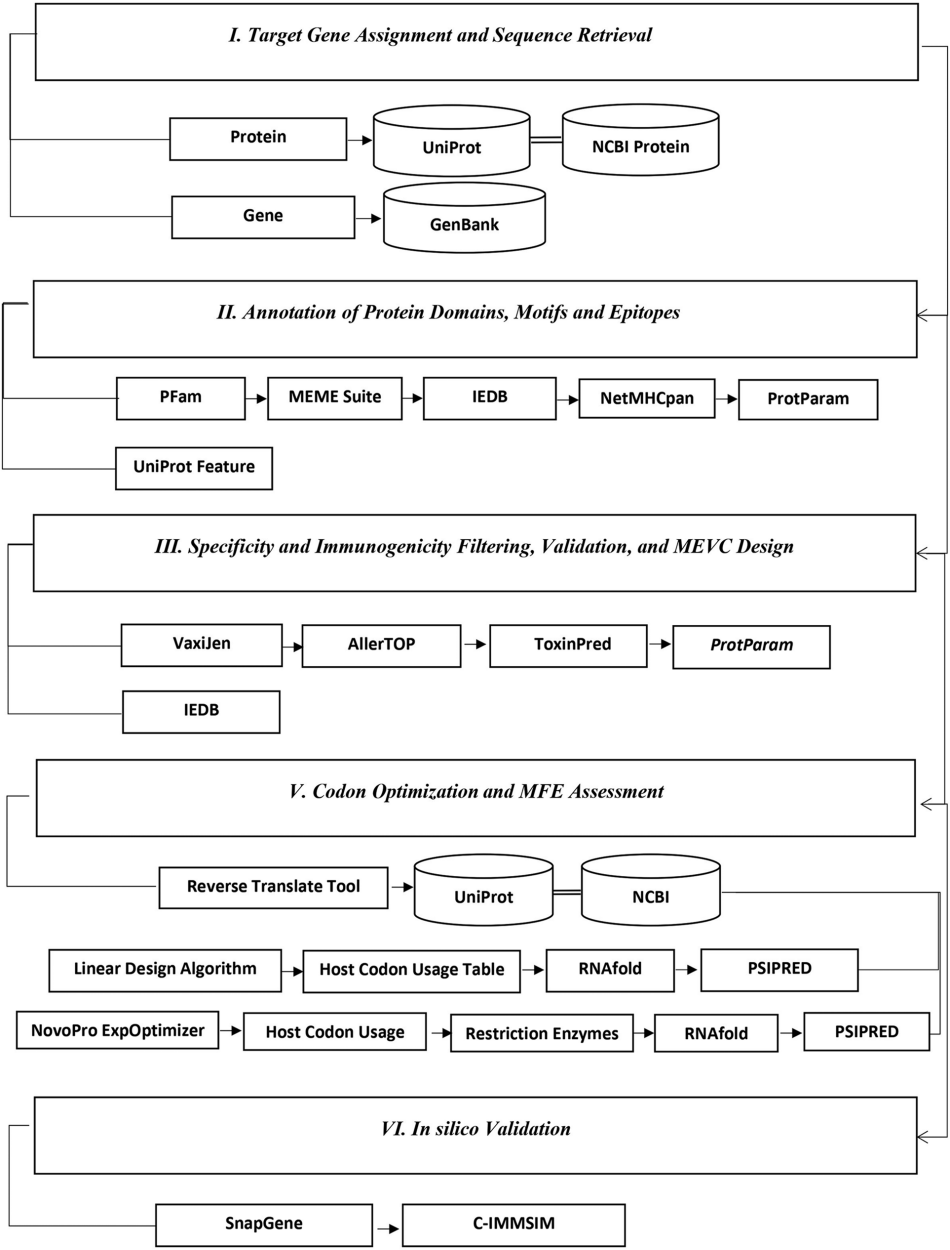
Procedure workflow for mRNA vaccine design. The figure outlines a multi-step pipeline for designing the multi-epitope vaccine construct (MEVC) and the full coding sequence (CDS), starting with target sequence retrieval from UniProt, NCBI protein, and GenBank. Protein domains, motifs, and epitopes are annotated and predicted using pfam, MEME suite, NetMHCpan, and IEDB. Predicted epitopes and the full-length CDS are evaluated for antigenicity, allergenicity, and toxicity using VaxiJen, AllerTOP, and ToxinPred, followed by physicochemical analysis with ProtParam. The MEVC is then constructed and re-evaluated for immunogenicity using the same tools. The coding sequence of MEVC is generated by reverse translation and optimized using LinearDesign and NovoPro ExpOptimizer, followed by RNAfold analysis for MFE and structural accessibility assessment then PSIPRED for 2D secondary structure prediction. Finally, validation with in silico cloning is performed using SnapGene, and immune simulation validation using C-ImmSim. Created with drawio.com

In this protocol, both approaches will be applied in parallel: full-length mRNA vaccines will be generated and evaluated, and top epitope sequences—selected for MEVC based on antigenicity, non-allergenicity, and experimental support—will be reverse-translated, optimized, tested and validated to design minimal yet effective mRNA vaccine candidates. To perform the procedure, multi-core CPUs (16–32 cores, as Intel Core i9) were used, with optional GPU acceleration, ≥16 GB RAM (64–128 GB recommended), and ≥1 TB SSD storage. The protocol was implemented on Linux-based systems, with support for macOS and Windows (via WSL2), and GPU-enabled applications required CUDA 11 +. The workflow integrates multiple bioinformatics tools and databases for sequence analysis and immunogenicity evaluation (Supplementary Table S2). To provide a structured overview, (Fig. 4) illustrates the complete workflow of the mRNA vaccine design procedure.

### Step 1: Target Gene Assignment and Sequence Retrieval

The first step is to obtain the target sequence, in this protocol, the spike protein of *SARS-CoV-2* (accession number P0DTC2). To retrieve it from the UniProt database (https://www.uniprot.org), search using the accession number and download the sequence in FASTA format. The same sequence can be obtained from the NCBI Protein database (https://www.ncbi.nlm.nih.gov) by selecting “Protein” in the search menu, entering the accession number, and downloading the FASTA file. If only the gene name is available, use NCBI or GenBank (https://www.ncbi.nlm.nih.gov/genbank/) to locate the corresponding protein entry by filtering results for the correct species, then obtain its accession number and FASTA sequence. This FASTA file will serve as the reference for all downstream optimization steps.

⚠ CRITICAL STEP: If the target protein is unknown, as in unannotated viral genomes, the entire genome is scanned to identify potential targets. Conserved regions across multiple strains are located using Clustal Omega (https://www.ebi.ac.uk/jdispatcher/msa/clustalo). Genome annotation is performed with Prokka (https://github.com/tseemann/prokka) to extract ORFs and protein-coding regions from the FASTA genome sequence. Predicted ORFs are translated via ExPASy Translate (https://web.expasy.org/translate/) and compared with viral protein databases (https://www.ncbi.nlm.nih.gov/labs/virus/) to exclude non-functional sequences, after which the downstream workflow pipeline is applied to generate the final vaccine sequence for both the MEVC and full length CDS.

### Step 2: Annotation of Protein Domains, Motifs and Epitopes

This step involves annotating the sequence to identify conserved domains, motifs, and potential epitopes using annotation and prediction tools, as outlined below:

#### Identify conserved protein domains and extracellular domains

Go to the Pfam website (https://pfam.xfam.org) and paste the P0DTC2 sequence then click on the “Search” tab. Pfam will then search its database of known protein domains and return a list of any matches found in the input sequence. For each match, Pfam provides detailed information about the domain, including its name, function, and location within the sequence. The output is a comprehensive annotation of protein domains within the spike protein, which can be used for further structural and functional analysis.

#### Discover conserved motifs

Open MEME Suite website (https://meme-suite.org) and select the “MEME” tool. Paste the P0DTC2 sequence and set the parameters based on known motif characteristics: 5–10 motifs, width 6–50 amino acids. This range captures key immunological and structural regions as short motifs (6–15 aa) often correspond to receptor binding domains or epitopes, while longer ones (20–50 aa) may represent structural domains like the fusion peptide or heptad repeats. Choose “zero or one occurrence per sequence” in protein mode to extract unique motifs, or “zero or more occurrences” to detect recurring motifs. Click “Submit”. MEME outputs statistically significant motifs with consensus sequences, highlighting conserved functional elements relevant to viral function and immune recognition.

#### MHC class I (CTL) and MHC class II (HTL) T cell epitope prediction

For MHC class I epitope prediction, open the IEDB (https://www.iedb.org/), select the T-cell epitope prediction tool (https://nextgen-tools.iedb.org/pipeline?tool=tc1) and input the sequence. Set all parameters to default and select a peptide length of 9 amino acids and choose the 27 human HLA class I alleles that provide approximately 97% population coverage. Run the prediction and export the results. For MHC class II epitope prediction, within the IEDB, open the tool (https://nextgen-tools.iedb.org/pipeline?tool=tc2), input the sequence and keep all parameters at default. Set the peptide length to 15 amino acids, and select the 27 human HLA class II alleles to ensure comparable population coverage. Run the prediction and export the results.

#### Linear/Confirmational B cell epitope prediction

For linear B-cell epitope prediction, within the IEDB open the tool (https://tools.iedb.org/bcell/) and input sequence selecting a prediction method such as BepiPred or Karplus–Schulz flexibility, which evaluates residue flexibility as an indicator of antigenicity. Run with default parameters. For conformational/discontinues B-cell epitope prediction, within IEDB, use ElliPro tool (https://tools.iedb.org/ellipro/) and input the validated 3D structure of the *SARS-CoV-2* spike protein (obtain it from the Protein Data Bank; https://www.rcsb.org/; PDB ID: 6VXX). In ElliPro, input the PDB ID and run with default parameters, including minimum score (protrusion index/PI threshold) required for a residue to be considered part of an epitope and maximum distance (Å) to define the clustering radius for grouping residues into epitopes. Submit the job, select all chains (A, B, and C), and resubmit to obtain results.

### Alternative method

**Fig. 5:**
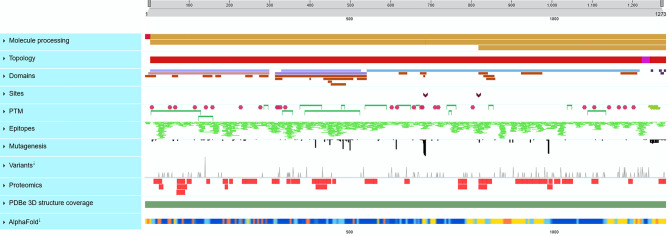
Visualization of SARS-CoV-2 spike protein annotations using the UniProt feature viewer. The UniProt feature viewer displays the spike protein sequence (1–1273), highlighting curated annotations including domains, motifs, topology (surface-exposed or extracellular regions of transmembrane proteins), and epitopes. Features are color-coded and mapped to sequence positions. GFF-formatted annotations with start/end positions and descriptions can be downloaded. A categorized panel allows selection of annotation layers such as molecular processing, topology, and structural features. Created with UniProt

Another annotation approach is using UniProt (https://www.uniprot.org), which provides curated spike protein annotations, including domains, motifs, topology (surface-exposed or extracellular regions of transmembrane proteins), and experimentally supported epitopes (Fig. 5). Search for accession number P0DTC2, open the entry, and navigate to the “Feature Viewer” tab to explore all features. Download the data via “Download” → “Features Only” → GFF format, which includes start/end positions, feature types, lengths, and descriptions (e.g., “Binding to ACE2 receptor”). This optional method provides curated, experimentally validated data, reduces prediction errors, and supports pipeline integration.

### Step 3: Specificity and Immunogenicity Filtering, Validation, and MEVC Design

#### MHC class I (CTL) and MHC class II (HTL) T cell filtering

For MHC class I/II epitopes, filter the results based on two main thresholds criteria. First, select epitopes with the lowest percentile rank (NetMHCpan EL percentile), as lower values indicate stronger binding affinity; consider values < 1% as strong binders and values between 1 and 2% as moderate binders. Second, select epitopes with the highest prediction score (NetMHCpan EL score), where values closer to 1 indicate stronger predicted binding affinity. If IC₅₀ information is available through the IEDB then a third optional filtration criteria can be applied, by selecting those with IC₅₀ less than 50 nM for very strong binders or less than 500 nM for average binders. IC₅₀ information could also be access through NetMHCpan server (https://services.healthtech.dtu.dk/services/NetMHCpan-4.1/) if not available in the IEDB.

#### *Linear/Confirmational B cell* epitope filtering

For linear B-cell epitopes, the tool assigns scores to residues or peptide segments, where higher scores indicate greater likelihood of antibody recognition. A threshold is automatically defined and region above it is considered potential epitopes. Select continuous high-scoring regions above the threshold as the most probable linear B-cell epitopes. For conformational/discontinues B-cell epitopes, the tool provides predicted epitopes with scores reflecting surface exposure and protrusion, where higher scores indicate greater accessibility. Select top-scoring epitopes and visualize them using the “View 3D structure” option to assess spatial distribution and surface accessibility.

#### IEDB assays validation

After filtering, validate the selected epitopes using the IEDB search interface by setting the host to “Human,” selecting both MHC class I and class II, then select all assay types (B-cell, T-cell, MHC Ligand) and filter for *SARS-CoV-2* spike protein epitopes. Select epitopes based on binding affinity and immune assays. Prioritize epitopes based on the number and type of associated experimental assays as T-cell activation and MHC binding assays, then review their ID to reference research articles to assess validation strength.

#### Validation of target specificity and immunogenicity

Assess sequences for antigenicity, toxicity, and allergenicity; if unsuitable, return to epitope selection. Use VaxiJen (threshold 0.4, “Virus” setting; http://www.ddg-pharmfac.net/vaxijen/VaxiJen/VaxiJen.html) to predict antigenicity scoring from FASTA sequences. Evaluate toxicity with ToxinPred (https://webs.iiitd.edu.in/raghava/toxinpred/) using “protein scanning” for full CDS, or the “Batch Submission” for the epitopes (excluding toxic peptides). Assess allergenicity using AllerTOP (https://www.ddg-pharmfac.net/AllerTOP/) by submitting FASTA sequences. Apply all evaluations to both selected epitopes and the full CDS.

#### Population coverage analysis

Set the number of epitopes according to the final selected list. Choose the query option *area_country_ethnicity* and select the targeted population (here we selected *World* to evaluate global population coverage). From MHC binding prediction results (NetMHC for class I and NetMHCII for class II), identify the most relevant HLA alleles for each epitope and select only the strongest binders based on binding affinity rank (Supplementary table S1), apply a strict cutoff of percentile rank ≤ 1.6. For each epitope, enter the sequence with its corresponding selected HLA alleles in the input table, ensuring correct matching. Select *Class I and II combined* to assess overall immune coverage, keep other parameters as default, and submit the analysis. After submission, review outputs including a graphical distribution of epitope recognition, population coverage percentage, average hit and PC90 that indicates the minimum number of epitope–HLA combinations recognized by 90% of the population.

#### *Multi-epitope mRNA* vaccine *construct*

Construct the multi-epitope vaccine by concatenating selected CTL, HTL, and B-cell epitopes into a continuous amino acid sequence using a biologically guided strategy, ensuring high binding affinity, antigenicity, non-allergenicity, and non-toxicity while avoiding redundancy. Begin with an immunostimulatory adjuvant at the N-terminus, such as human β-defensin 2 (HBD2), retrieve its sequence from (https://www.rcsb.org/structure/1FD3), followed by a rigid EAAAK linker to maintain structural separation. Arrange epitopes in a defined order: HTL epitopes first (linked by GPGPG) to support MHC class II presentation, followed by CTL epitopes (AAY) to enhance proteasomal processing and MHC class I presentation, and finally B-cell epitopes (KK) to preserve antibody accessibility. Assemble the construct as: adjuvant → EAAAK → HTL (GPGPG) → CTL (AAY) → B-cell (KK). Verify sequence continuity, correct reading frame, and absence of premature stop codons, and evaluate the construct as a single protein. Although simplified designs without linkers are possible, rational linker-based assembly is preferred; thus, the MEVC strategy was applied throughout this protocol.

To assess immunogenicity of the MEVC sequence, use the same tools as in the previous step: VaxiJen for antigenicity, AllerTOP for allergenicity, and ToxinPred for toxicity. In addition, use ProtParam (https://web.expasy.org/protparam/) to evaluate physicochemical properties of MEVC. Paste the MEVC sequence and submit. Check from the output, the molecular weight, stability, hydrophobicity, secondary structure tendencies, and in vivo and in vitro half-life, to confirm structural stability and suitability for expression. Verify that linker inclusion does not disrupt epitope integrity or reduce predicted binding affinity. In this protocol, five epitopes were selected from each category (MHC class I, MHC class II, linear B-cell, and conformational B-cell). Initial filtering was based on high binding affinity (percentile rank ≤ 1.6) and highest prediction scores, with antigenicity assessed only for this subset. Final selection considered immunogenicity, antigenicity, and validation in IEDB assays. Linear B-cell epitopes were further concatenated in pairs to enhance antigenic coverage and potentially strengthen humoral responses, consistent with validated IEDB regions. Although optional, this step was applied to improve epitope representation and immunogenic potential.

### Step 4: Codon Optimization and MFE Assessment

First, retrieve the CDS for the selected protein or epitopes/MEVC. Download directly from databases when available; otherwise, generate it using a reverse translation tool. For *SARS-CoV-2* spike, obtain accession P0DTC2 from UniProt and follow cross-references to the GenBank record (NC_045512.2), where the “surface glycoprotein” CDS (21563– 25,384) can be downloaded in FASTA format. Alternatively, use NCBI (Gene ID: 43,740,568 or NC_045512.2) to retrieve the CDS via genome annotation tools. Both full-length CDS and MEVC-based sequences are used in parallel.

The following step involves codon optimization of the CDS or MEVC to enhance translational efficiency. Use the *LinearDesign* algorithm (https://github.com/LinearDesignSoftware/LinearDesign, command-line version) to calculate CAI and MFE, running the CDS with λ = 3, which favors translation efficiency while maintaining structural stability (λ = 0 prioritizes structure; λ = 1–2 balances both; λ >3 biases codon usage). Optimize the codon usage to match the host organism’s preference, by selecting human as a host. After optimization, append UTRs and evaluate the full mRNA using RNAfold (http://rna.tbi.univie.ac.at//cgi-bin/RNAWebSuite/RNAfold.cgi) to assess MFE and secondary structure stability. In this protocol, *LinearDesign* was applied to the spike CDS (YP_009724390.1; Gene ID 43,740,568) and subsequently to the MEVC. Alternatively, using NovoPro ExpOptimizer tool (https://www.novoprolabs.com/tools/codon-optimization), input the MEVC amino acid sequence, select protein as input type, and choose *Human* and select restriction sites to be avoided (e.g., BamHI, XhoI) to prevent internal cleavage during cloning. Multiple runs may be performed to obtain the most optimized sequence. NovoPro provides CAI and GC content but not MFE; therefore, RNAfold was used for structural evaluation. Both CDS and MEVC were optimized, with emphasis on MEVC for cloning.

⚠ CRITICAL STEP: Target accessibility assessment ensures that key mRNA regions, especially around ribosome binding sites and epitopes, remain structurally accessible for translation and immune recognition. Evaluate optimized CDS and MEVC sequences using ViennaRNA tools (http://rna.tbi.univie.ac.at/): RNAfold for MFE secondary structure and accessibility, RNAeval for thermodynamic analysis, and RNAplfold for local base-pairing probabilities. As optional validation, analyze the MEVC at the protein level using PSIPRED (http://bioinf.cs.ucl.ac.uk/psipred/) for secondary structure prediction, perform molecular docking with immune receptors (e.g., TLR4) using ClusPro (https://cluspro.org/) with visualization in PyMOL (https://pymol.org/), and assess complex stability via molecular dynamics simulations using GROMACS (http://www.gromacs.org/). In this protocol, an additional step was performed for 2D structure analysis using PSIPRED to predict the coil structure, alpha-helix, and beta-sheet of the optimized MEVC. To perform that, open PSIPRED, choose to predict secondary structure, translate the optimized MEVC DNA sequence (resulted by NovoPro) into protein by Expasy translate tool (https://web.expasy.org/translate/) to input the optimized MEVC sequence and run the analysis.

### Step 5: In silico Validation

To verify cloning and expression feasibility of the optimized mRNA construct perform in silico cloning, starting with selecting a suitable expression vector such as pET-28a(+), and download its sequence from Addgene (https://www.addgene.org), in SnapGene format. Import the vector into SnapGene (https://www.snapgene.com/) and review its annotated features. Open the optimized insert MEVC separately and perform restriction analysis on both vector and insert. Select BamHI (GGATCC) and XhoI (CTCGAG) based on their presence in the MCS, ability to enable directional cloning, and absence of internal sites in the insert. Add BamHI to the 5′ end and XhoI to the 3′ end of the CDS. Simulate digestion of both vector and insert with BamHI and XhoI to generate compatible ends. To perform this, open the vector file in SnapGene and select “Actions → Restriction and Insertion Cloning → Insert Fragment” to ligate the modified MEVC insert. Cut both the vector and insert with BamHI and XhoI then flip the fragment orientation if required to ensure correct directional insertion, as indicated by SnapGene orientation warnings. Validate the construct by confirming ORF continuity, correct start codon positioning, absence of internal stop codons, and the expected total plasmid size (~5.3 kb + insert).

For Immune simulation validation, evaluate the immunogenic potential of the designed MEVC using the C-IMMSIM server (https://kraken.iac.rm.cnr.it/C-IMMSIM/). Input the final amino acid sequence into the submission field then run the simulation using default parameters unless specific conditions are required. Define simulation steps to represent vaccine doses as single or multiple injections by defining time intervals corresponding to primary and booster immunizations. In this protocol, a single-dose injection model was used. Configure simulation volume and random seed if needed, then run the analysis. The platform uses PSSM and machine learning to model immune responses, including antigen processing and immune cell activation. Analyze outputs such as immunoglobulin levels (IgM, IgG), B- and T-cell populations, cytokine profiles (as IFN-γ, IL-2), and memory cell formation. A strong response is indicated by elevated antibody levels, robust T-cell activation, and sustained memory development.

⚠ CRITICAL STEP: Following the main workflow, optional UTR and CDS/MEVC optimization can be performed for experimental readiness. This involves testing combinations of 5′ and 3′ UTRs with the CDS or MEVC (total = number of 5′ UTRs × number of 3′ UTRs). UTRs are retrieved from UTRdb (https://utrdb.cloud.ba.infn.it/utrdb/index_107.html) or Ensembl (https://www.ensembl.org), combined with the CDS/MEVC (5′ UTR + CDS/MEVC + 3′ UTR), and analyzed using RNAfold (http://rna.tbi.univie.ac.at/cgi-bin/RNAWebSuite/RNAfold.cgi) to obtain MFE, secondary structure, and base-pair probabilities, where lower MFE indicates higher stability. For MEVC, reverse translation (https://www.bioinformatics.org/sms2/rev_trans.html) and mRNA transcription using Biomodel (https://biomodel.uah.es/en/lab/cybertory/analysis/trans.htm) are performed before UTR integration. For human expression, validated UTR pairs such as TMSB10 (5′) with APOA2 or AP3B1 (3′) are prioritized. An additional optional step evaluates nucleotide modifications, such as pseudouridine, using RNAfold energy corrections to assess their effects on structure and stability.

## Results and discussion

**Fig. 6:**
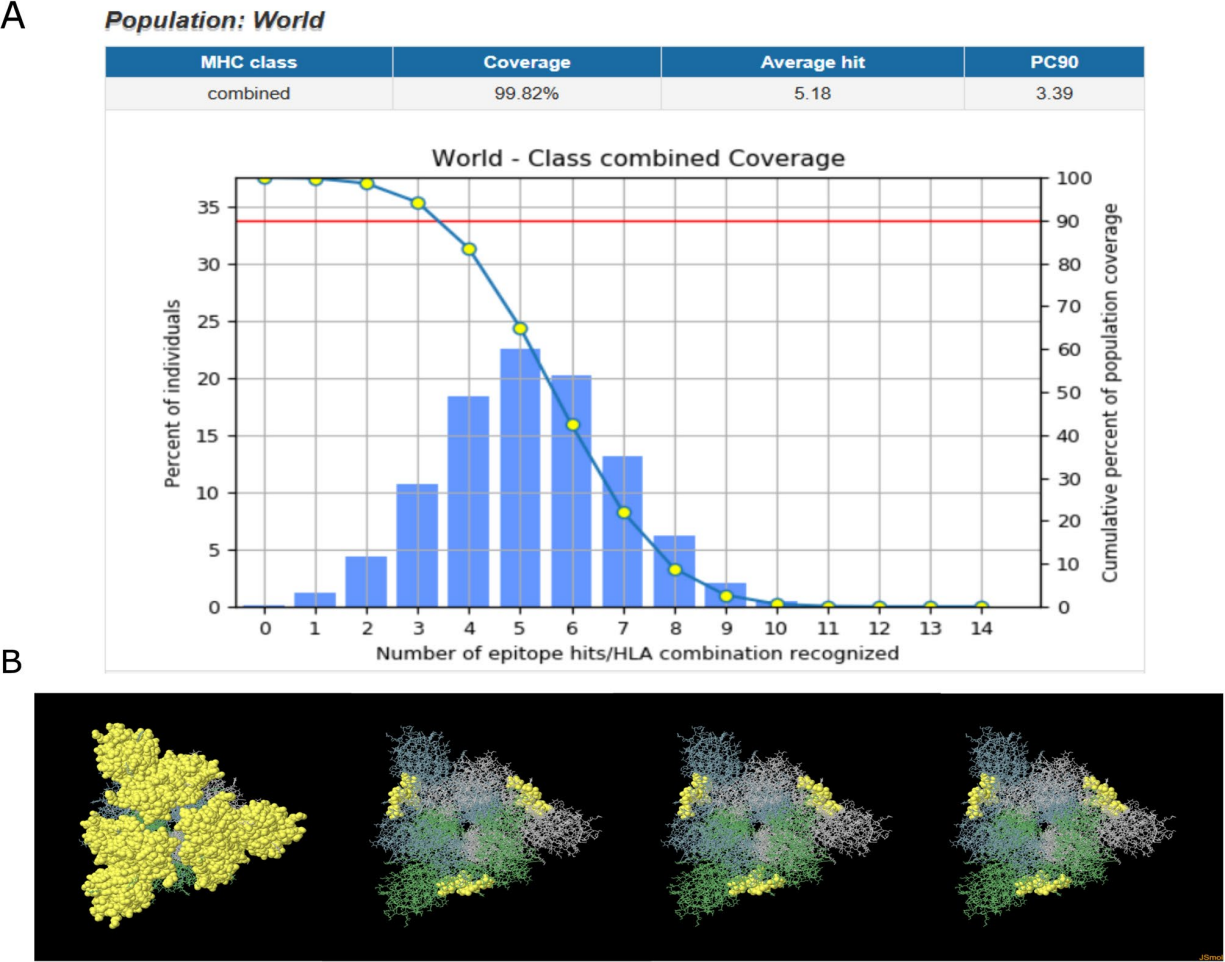
Population coverage and conformational B-cell epitope mapping. (**A**) global world population coverage analysis of the selected epitopes based on HLA binding, showing high worldwide coverage, average hit, and PC90 values, indicating broad population inclusivity and the ability of most individuals to recognize multiple epitope–HLA combinations. Created with IEDB/Population. (**B**) structural mapping of predicted conformational B-cell epitopes on the SARS-CoV-2 spike protein, where yellow highlights indicate epitope residues. The first panel corresponds to the top-ranked epitope spanning chains A and C (Supplementary table S1), whereas the remaining panels represent selected epitopes mapped on chains A, B, and C, respectively. Created with IEDB/Ellipro

In this work, we present a comprehensive step-by-step protocol for the design of mRNA vaccines targeting both full-length coding sequence (CDS) and multi-epitope vaccine construct (MEVC) approaches. This protocol was applied to the *SARS-CoV-2* spike protein (UniProt: P0DTC2) as the vaccine target due to its critical role in viral entry, its conservation across strains, and its well-established immunogenicity supported by extensive structural and immunological evidence [85–91]. Our comprehensive epitope profiling revealed a dense landscape of predicted T- and B-cell epitopes. Specifically, we identified 29,620 and 5,671 MHC class I and MHC class II T-cell peptides respectively, as well as 1,266 linear and 10 conformational B-cell epitopes. To identify the epitopes suitable for MEVC design, these epitopes were further ranked, and the top 1.6 percentile sequences were shortlisted, as indicated in the procedures, resulting in 1,443 MHC class I, 99 MHC class II epitopes. From these T-cell and B-cell identified epitopes, we removed epitopes that exhibited toxicity, allergenicity and non-antigenicity, as detailed in the procedure. The remaining epitopes were sorted based on the antigenicity score, binding affinity, prediction confidence and the IEDB assays, and the top five epitopes of MHC class I, MHC class II, and B-cell linear as well as conformational peptides were selected. Additionally, 4 top-scoring conformational B-cell epitopes were identified as surface-accessible regions (Fig. 6B) and used to validate the antigenic accessibility of the MEVC (Supplementary Table S1). The selected CTL and HTL epitopes have been independently validated to elicit CD8^+^ and CD4^+^ T-cell responses in infected or vaccinated individuals [92–99], while B-cell epitopes mapped to structurally exposed and antibody-accessible regions, including the receptor-binding domain (RBD) and S1 subunit [90, 91, 99–103].

Antigenicity assessment of the full-length CDS demonstrated a high antigenicity score of 0.47, while the MEVC exhibited a markedly higher antigenicity score of 1.70, supporting the effectiveness of multi-epitope vaccine design strategies in enhancing predicted immunogenic potential [104]. Toxicity screening of the CDS identified only three short peptide fragments as potentially toxic, namely VCGPKKSTNLVKNKC, CGPKKSTNLVKNKCV, and GPKKSTNLVKNKCVN; however, these fragments were already excluded from the final construct of MEVC, reflecting an improved safety profile. Physicochemical analysis of the MEVC (309 amino acids; 33.74 kDa) indicated favorable properties, including stability (instability index: 26.94 and >30 h half-life in human cells), thermostability (aliphatic index: 74.24), and hydrophilicity (GRAVY: −0.146, indicating a hydrophilic nature and enhanced solubility), along with suitable expression stability across systems.

Population coverage analysis demonstrated near-universal representation across diverse HLA alleles, with multiple epitope–HLA interactions per individual. Specifically, population coverage reached 99.82%, with an average hit of 5.18 and a PC90 value of 3.39, indicating that at least 90% of the population can recognize approximately 3–4 epitopes through HLA combinations, providing redundancy in immune recognition and reducing the likelihood of immune escape [105]. The distribution of 4–6 recognized epitopes per individual and a cumulative coverage approaching 100% further support robust and inclusive immune coverage (Fig. 6A). The findings support the suitability of the selected epitopes for multi-epitope vaccine design, as they maximize immune coverage across diverse HLA genotypes with redundancy in antigen recognition.

**Fig. 7:**
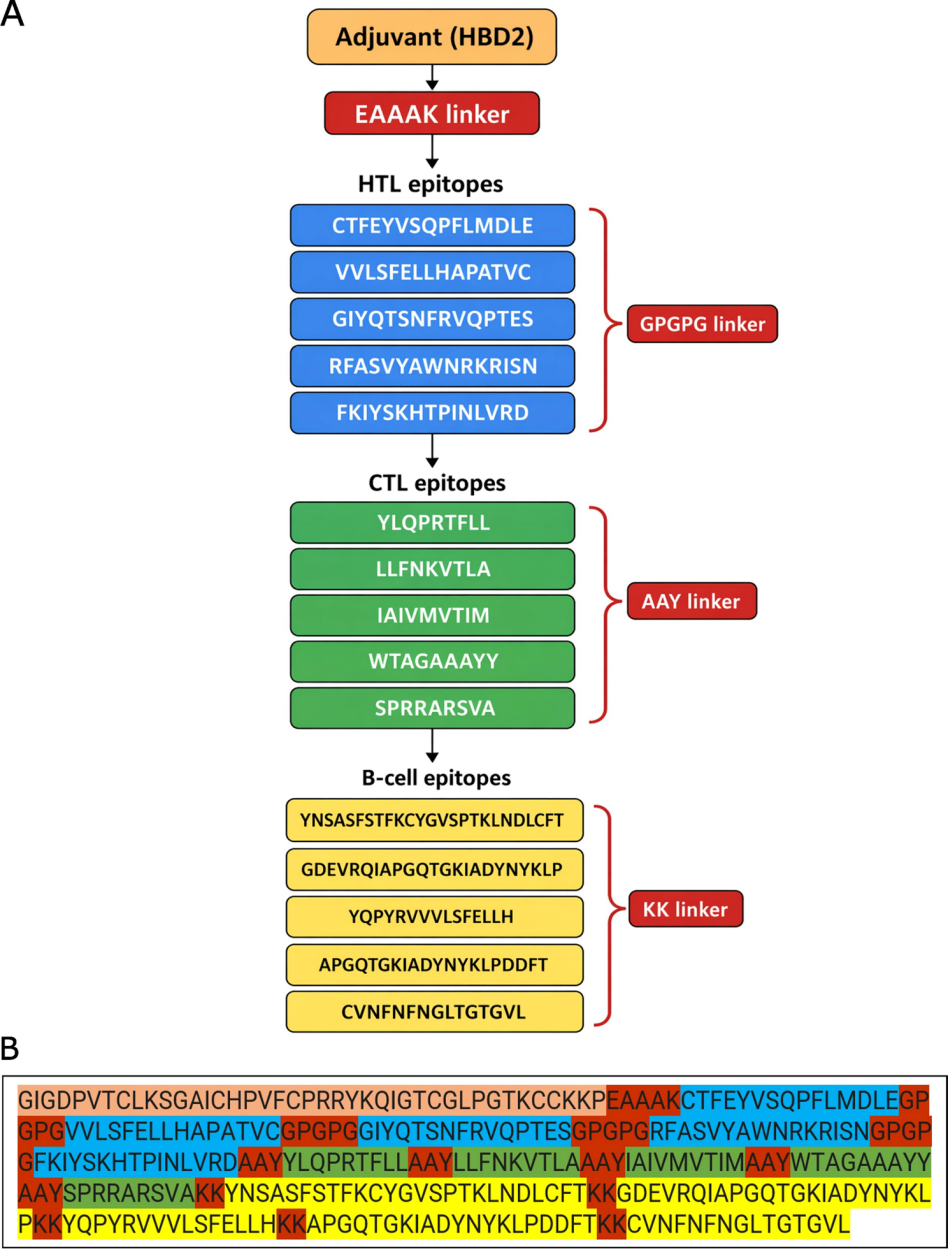
Design of the multi-epitope vaccine construct (MEVC). (**A**) schematic representation of the arrangement of adjuvant (HBD2), linkers, and selected HTL, CTL, and B-cell epitopes. (**B**) final amino acid sequence of the MEVC showing the concatenated epitopes and linker regions. Color coding: adjuvant (orange), HTL epitopes (blue), CTL epitopes (green), B-cell epitopes (yellow), and linkers (red). Created with drawio.com and MS word

The final MEVC was assembled using a rational design strategy, placing HTL epitopes first, followed by CTL and B-cell epitopes, connected by GPGPG, AAY, and KK linkers, respectively (Fig. 7A–B). These linkers are experimentally supported for enhancing antigen processing and presentation [46, 106, 107]. Human β-defensin 2 (HBD2) was incorporated as an adjuvant at the N-terminus via an EAAAK linker, consistent with studies demonstrating its role in activating dendritic cells and promoting cytokine responses through TLR pathways [48, 49, 103]. The resulting design formed a continuous open reading frame without frame shifts or premature stop codons, confirming structural integrity.

**Fig. 8:**
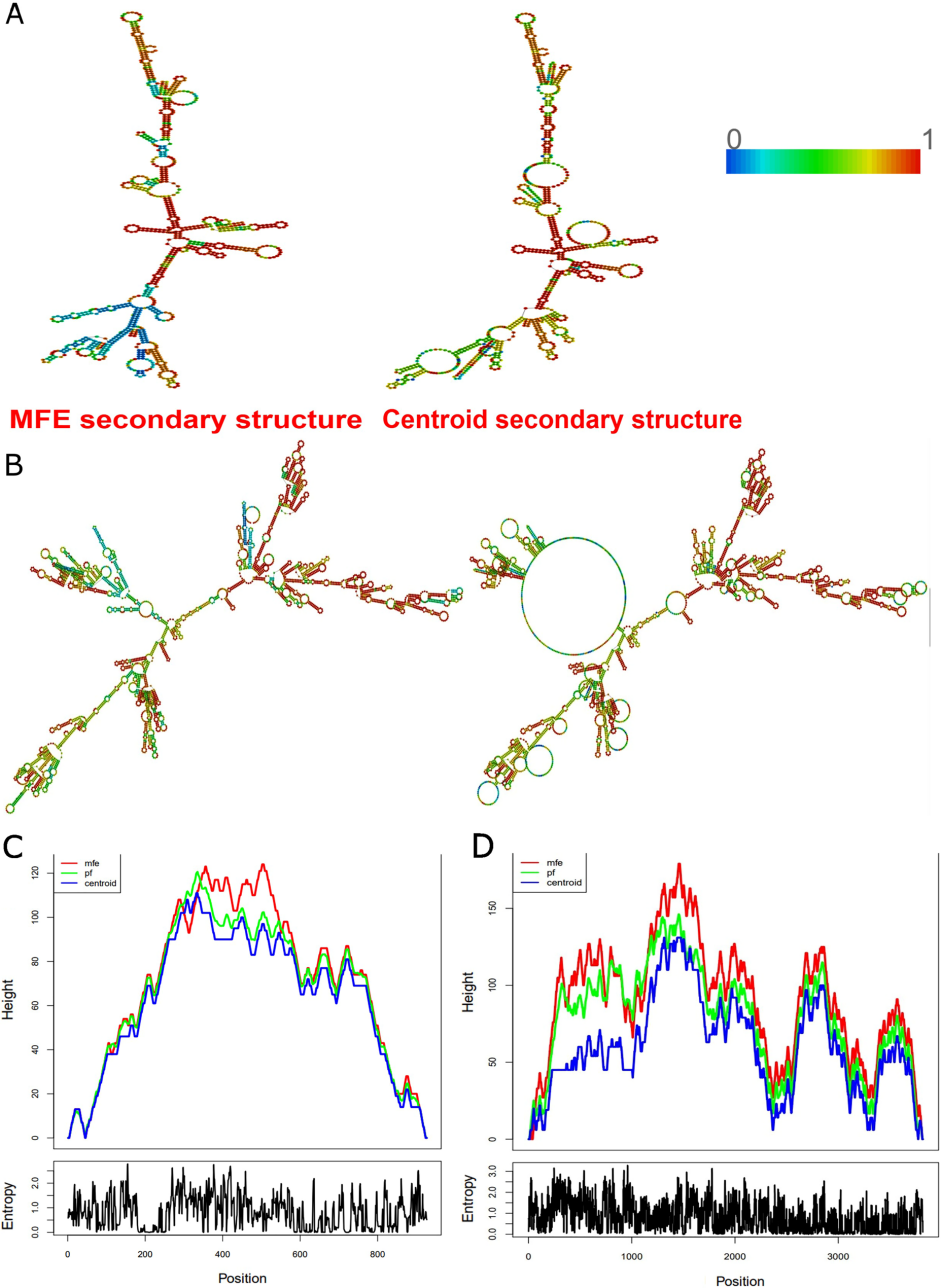
RNA secondary structure analysis of optimized MEVC and full-length CDS. (**A**) predicted secondary structures of the optimized MEVC, where the minimum free energy (MFE) structure is shown on the left and the centroid structure on the right. (**B**) predicted secondary structures of the optimized full-length CDS, with the MFE structure on the left and the centroid structure on the right, illustrating increased structural complexity due to the longer sequence length. In both (**A**) and (**B**), structures are colored according to base-pairing probabilities (0–1 scale), where red indicates highly stable paired regions and blue/green indicates lower pairing probability and higher accessibility. (**C**) mountain plot representation of the MEVC showing MFE (red), partition function (green), and centroid (blue) profiles, indicating stable folding behavior with a balance between structured and accessible regions. (**D**) mountain plot of the full-length CDS displaying similar trends with greater structural variation, reflecting its extended length. Created with RNAfold

**Fig. 9:**
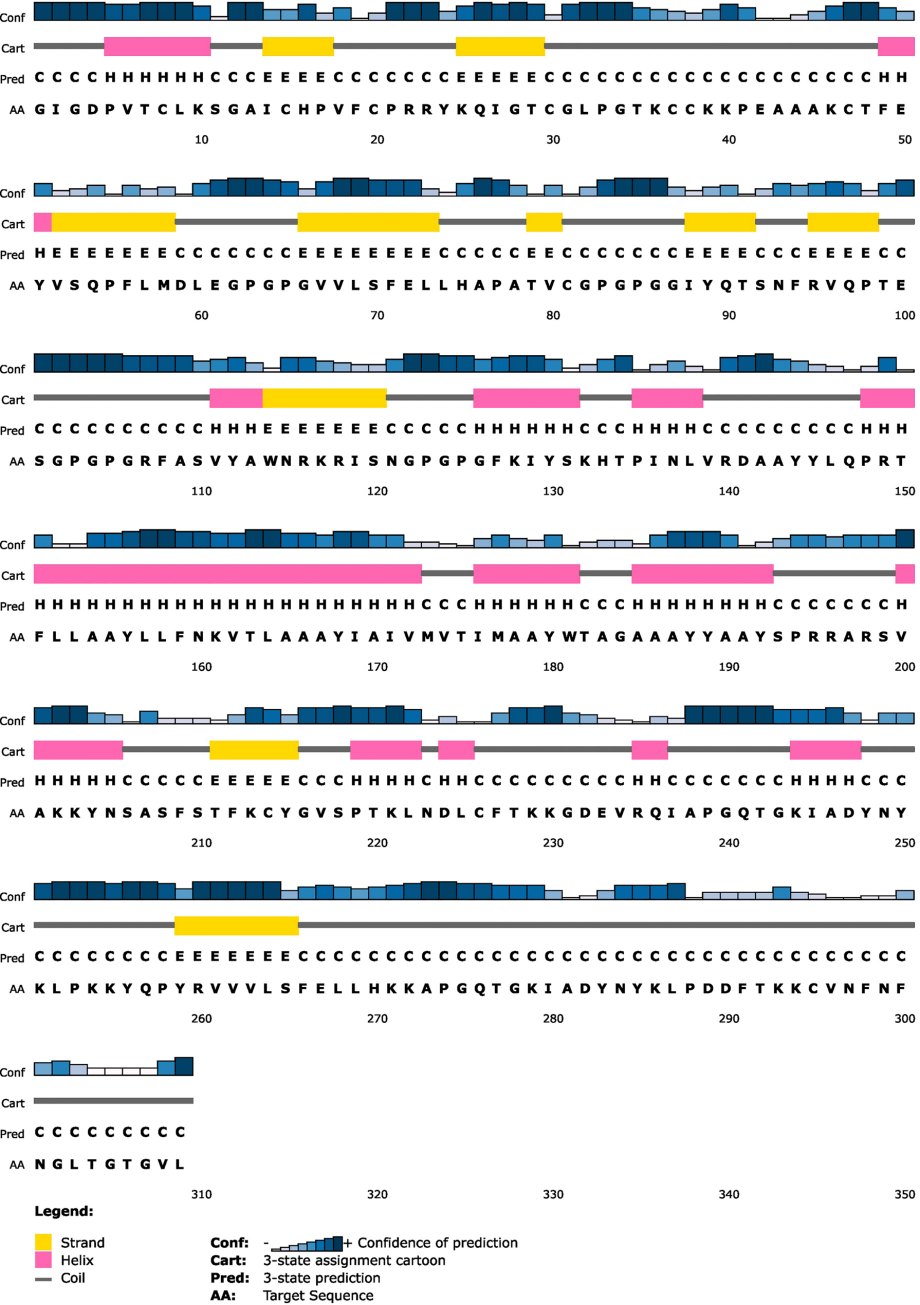
Secondary structure (2D) prediction of the optimized MEVC. The figure illustrates the predicted secondary structural elements along the protein sequence, including α-helices (pink), β-sheets (yellow), and coil regions (gray). The upper panel represents the confidence score for each amino acid residue, where darker shades indicate higher prediction confidence. The distribution of structural elements shows the presence of both ordered regions (α-helices and β-sheets) and flexible coil regions, supporting the structural stability and functional flexibility of the optimized MEVC. Created with PSIPRED

Codon optimization of the full-length CDS and designed MEVC yielded optimal CAI values of 0.81 and 0.83 with GC contents of 50.35% and 53.61%, respectively, indicating strong adaptation to human expression systems. These results are consistent with experimental evidence demonstrating that codon optimization enhances recombinant protein expression [11, 108, 109]. On the other hand, RNA secondary structure analysis revealed stable mRNA conformations with highly negative MFE values of −1231.20 kcal/mol for the optimized CDS, reflecting its longer sequence (3819 nucleotides, 1273 amino acids), and MFE of −299.50 kcal/mol for the optimized MEVC (927 nucleotides, 309 amino acids). The predicted structures exhibit stem–loop formations that maintain a balance between structural stability and accessibility (Fig. 8A–D), while preserving regions of accessibility required for efficient ribosome binding [110]. This aligns with previous findings that a balance between structural stability and accessibility (e.g., moderate entropy) enhances ribosome binding and sustained expression [30, 91, 111]. For additional extra analysis, the predicted 2D secondary structure of the optimized MEVC showed a heterogeneous distribution of α-helices, β-sheets, and predominantly coil regions, indicating structural flexibility with localized stable motifs. High confidence scores across most residues suggest reliable predictions (Fig. 9).

**Fig. 10:**
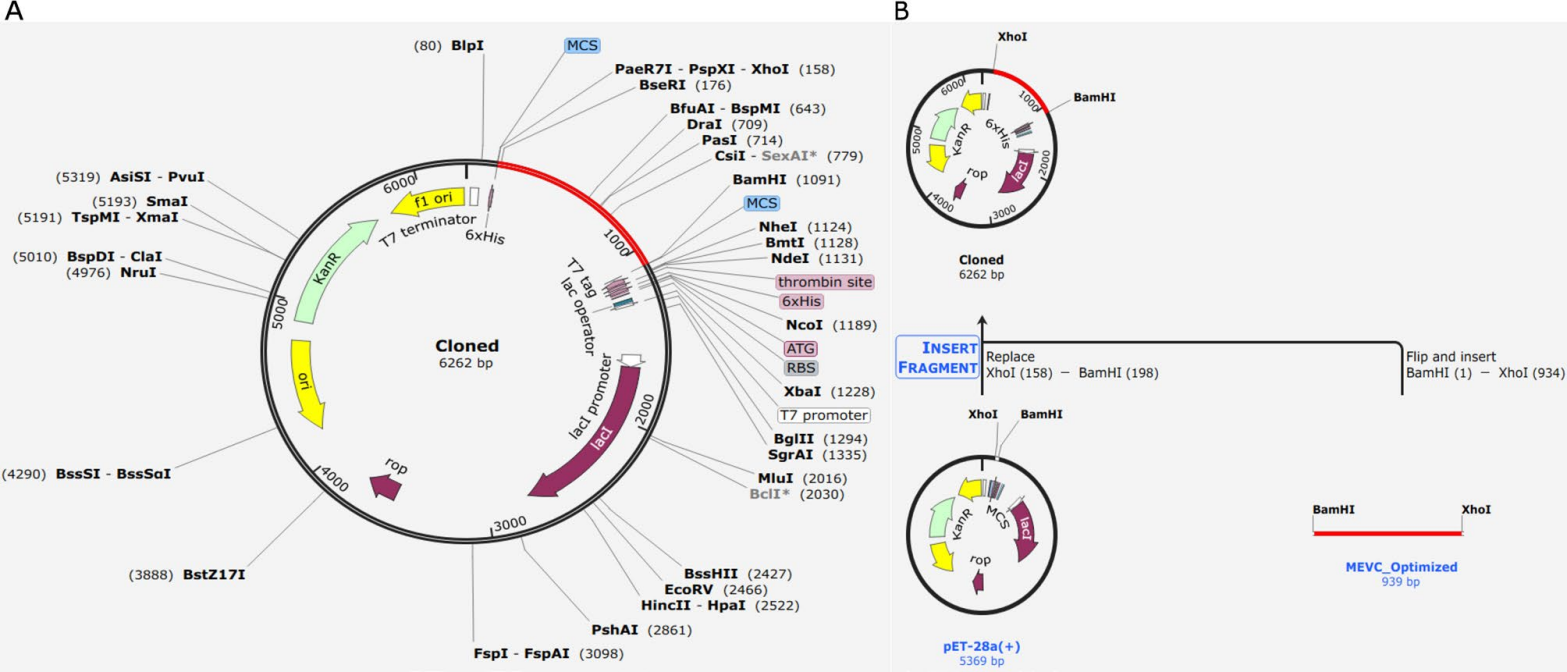
In silico cloning of the MEVC into the pET-28a(+) vector. (**A**) in silico cloning of the adapted vaccine sequence into the pET-28a(+) expression vector, showing the selected cloning region highlighted in red between XhoI (158) at the N-terminal and BamHI (1091) at the C-terminal, while the vector backbone is represented in black. (**B**) cloning construction workflow illustrating the insertion of the optimized MEVC fragment (939 bp) into the pET-28a(+) vector (5369 bp), resulting in a recombinant plasmid of 6262 bp. Directional cloning using BamHI (C-terminal) and XhoI (N-terminal) ensures correct orientation of the insert within the vector. Created with SnapGene

In silico cloning confirmed the successful integration of the MEVC into the pET-28a (+) expression vector, enabling directional cloning and correct orientation relative to the T7 promoter (Fig. 10A–B). The final construct preserved all essential vector elements, including the ribosome binding site, start codon, His-tag, kanamycin resistance gene, and origin of replication. The absence of frame shifts or sequence disruptions confirms the structural integrity of the recombinant plasmid and supports its suitability for recombinant protein production and preliminary experimental validation. The compatibility of the construct with a widely used expression vector further supports its translational feasibility [62–64, 112].

**Fig. 11:**
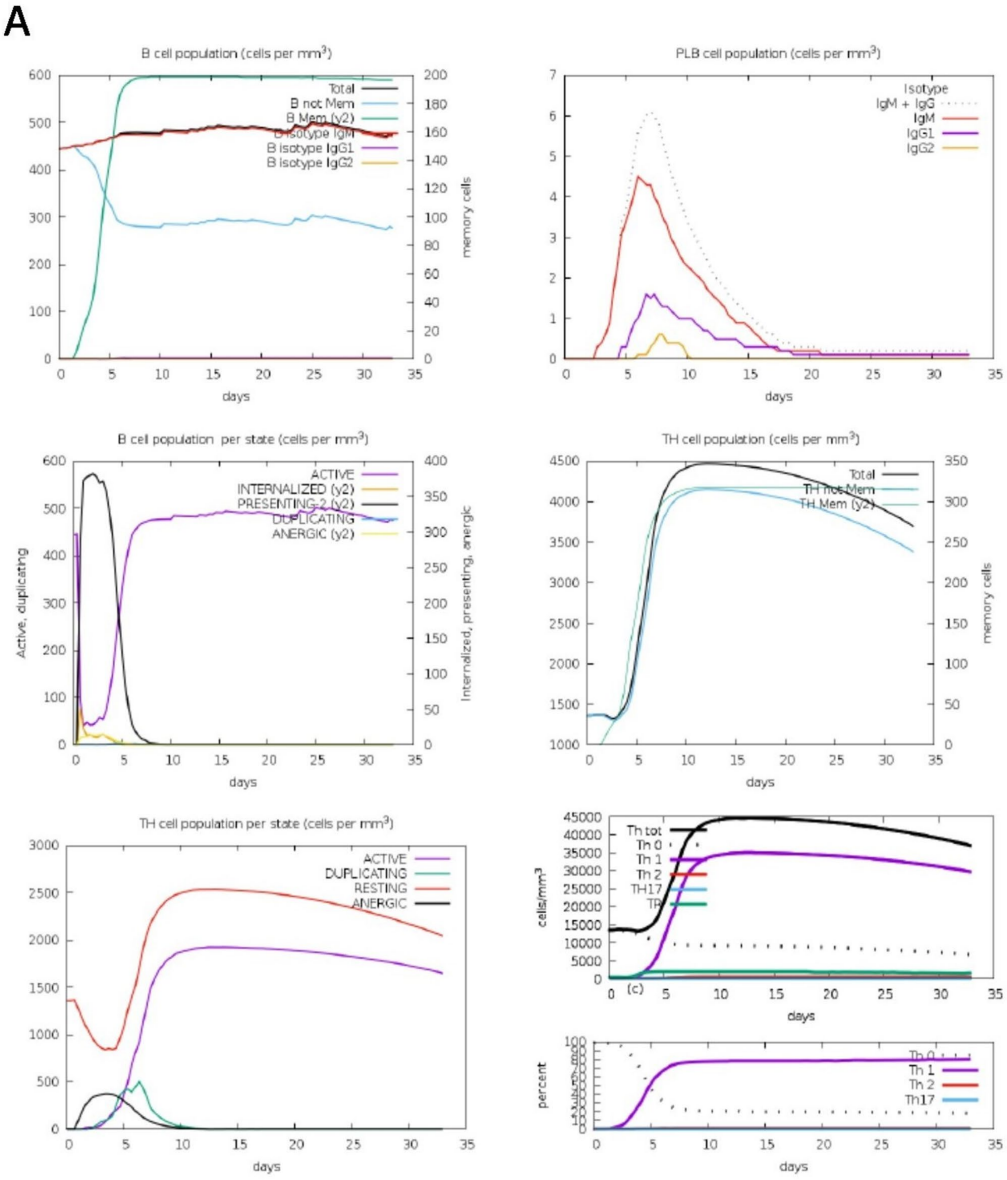

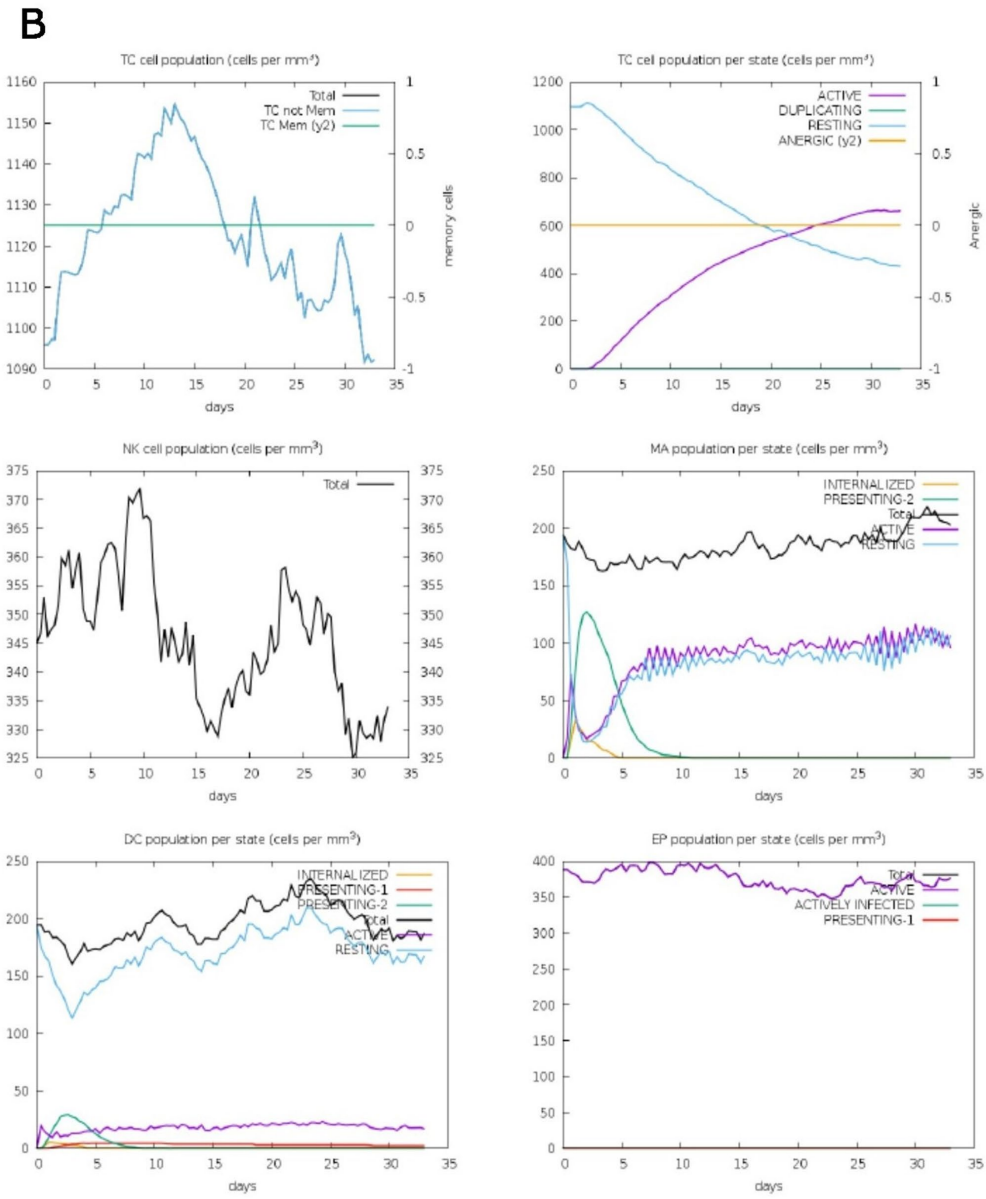

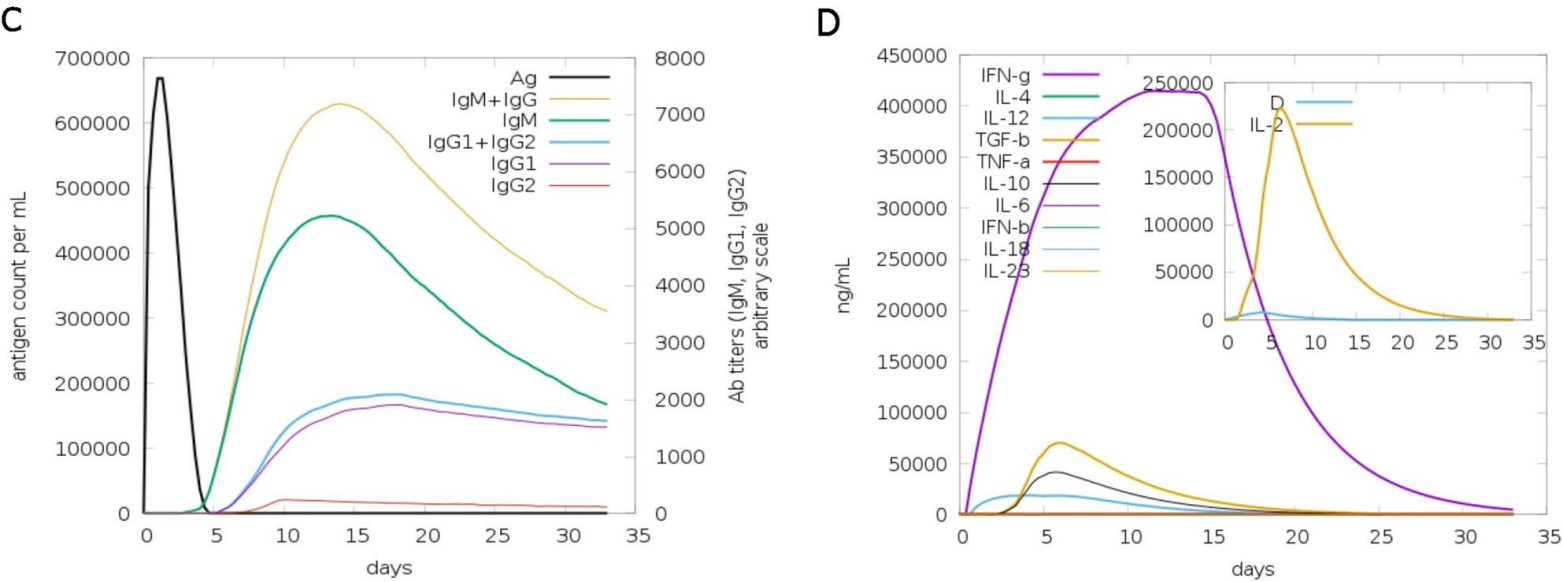
Immune simulation results of the optimized designed MEVC. (**A**) B-cell and plasma cell dynamics, including total B-cell count, memory B cells, immunoglobulin isotypes (IgM, IgG1, IgG2), and B-cell population states (active, presenting, duplicating, and anergic), illustrating activation, proliferation, differentiation, and memory formation following antigen exposure. (**B**) cellular immune response and innate immune dynamics, including total T-cell populations (helper and cytotoxic T cells), T-cell memory subsets, natural killer (NK) cells, dendritic cells (DCs), macrophages, and epithelial cells, demonstrating coordinated immune activation, antigen presentation, and early innate responses. (**C**) antigen and antibody profile, showing rapid antigen (ag) clearance accompanied by a typical primary immune response with IgM production followed by class switching to IgG subclasses (IgG1 and IgG2), indicating effective humoral immunity. (**D**) cytokine and interleukin response, highlighting elevated levels of IFN-γ, IL-2, and other key cytokines (e.g., IL-4, IL-6, TNF-α), reflecting strong immune activation and regulation of cellular immune responses. Created with C-IMMSIM

Immune simulation using a single-dose injection model demonstrated a strong primary immune response (Fig. 11A–D), characterized by rapid B-cell expansion, memory cell formation, and activation of HTL and CTL represented in the dynamic cellular profiles. Antibody profiles showed typical IgM-to-IgG class switching pattern accompanied by antigen clearance, indicating effective humoral immunity. Elevated cytokine levels, particularly IFN-γ and IL-2, further reflected robust immune activation and coordination between immune cell populations. These results align with prior studies showing that in silico immune simulations can recapitulate key features of experimentally observed immune responses, supporting their utility as an early validation steps [99, 103, 113, 114].

The time required to execute this procedure for a target protein such as the *SARS-CoV-2* spike protein (P0DTC2), was approximately 2–3 hours, depending on server availability and computational load. In contrast, analysis of unknown targets typically requires additional computational steps and longer processing time. Therefore, the development of automated software solutions would be valuable to streamline and optimize mRNA vaccine design workflows, particularly for emerging pathogens where rapid response is critical.

## Conclusion

This protocol establishes a comprehensive computational framework for mRNA vaccine design by integrating immunoinformatics, structural biology, and molecular optimization into a unified workflow. The pipeline ensures efficient antigen selection, stable mRNA design, and robust immune activation. The protocol defines the design space, key selection criteria, and stepwise procedures required for the development of both full-length CDS and MEVC, which represent a reproducible and adaptable strategy applicable to different pathogens, particularly in scenarios requiring rapid vaccine development. The application of this framework to the *SARS-CoV-2* spike protein demonstrated its ability to generate constructs with high antigenicity, broad population coverage, optimized codon usage, and stable RNA secondary structures, supported by in silico cloning and immune simulation validation analyses of the MEVC. These results are consistent with experimentally validated findings and highlight the effectiveness of combining epitope-driven design with sequence-level optimization strategies. While computational predictions indicate strong immunogenic potential and translational feasibility, further in vitro and in vivo studies are necessary to validate the safety, efficacy, and protective performance of the designed construct.

## Electronic supplementary material

Below is the link to the electronic supplementary material.

Supplementary material 1

Supplementary material 2

## References

[1] Boccalini S. Value of vaccinations: A fundamental public health priority to be fully evaluated. Vaccines. Multidisciplinary Digital Publishing Institute (Mdpi); 2025;13:479. 10.3390/vaccines13050479. 40432091 10.3390/vaccines13050479 PMC12115698

[2] Andre F, Booy R, Bock H, Clemens J, Datta S, John T, et al. Vaccination greatly reduces disease, disability, death and inequity worldwide. Bull World Health Organ. 2008;86:140–6. 10.2471/blt.07.040089. 18297169 10.2471/BLT.07.040089 PMC2647387

[3] Maruggi G, Zhang C, Li J, Ulmer JB, Yu D. mRNA as a transformative technology for vaccine development to control infectious diseases. Mol Ther. 2019;27:757–72. 10.1016/j.ymthe.2019.01.020. 30803823 10.1016/j.ymthe.2019.01.020 PMC6453507

[4] Pardi N, Hogan MJ, Porter FW, Weissman D. mRNA vaccines - a new era in vaccinology. Nat Rev Drug Discov. 2018;17:261–79. 10.1038/nrd.2017.243. 29326426 10.1038/nrd.2017.243 PMC5906799

[5] Sahin U, Karikó K, Türeci Ö. mRNA-based therapeutics--developing a new class of drugs. Nat Rev Drug Discov. 2014;13:759–80. 10.1038/nrd4278. 25233993 10.1038/nrd4278

[6] Verbeke R, Lentacker I, De Smedt SC, Dewitte H. The dawn of mRNA vaccines: The COVID-19 case. J Control Release. 2021;333:511–20. 10.1016/j.jconrel.2021.03.043. 10.1016/j.jconrel.2021.03.043 PMC8008785 33798667

[7] Walsh EE, Frenck RW, Falsey AR, Kitchin N, Absalon J, Gurtman A, et al. Safety and immunogenicity of two RNA-based Covid-19 vaccine candidates. N Engl J Med. 2020;383:2439–50. 10.1056/NEJMoa2027906. 10.1056/NEJMoa2027906 PMC7583697 33053279

[8] Verbeke R, Hogan MJ, Loré K, Pardi N. Innate immune mechanisms of mRNA vaccines. Immun. 2022;55:1993–2005. 10.1016/j.immuni.2022.10.014. 36351374 10.1016/j.immuni.2022.10.014 PMC9641982

[9] Leong KY, Tham SK, Poh CL. Revolutionizing immunization: a comprehensive review of mRNA vaccine technology and applications. J Virol. 2025;22:71. 10.1186/s12985-025-02645-6. 40075519 10.1186/s12985-025-02645-6 PMC11900334

[10] Jarzebska NT, Frei J, Mellett M, Kündig TM, Pascolo S, Reichmuth AM. A basic method for formulating mRNA-lipid nanoparticle vaccines in the lab. Methods in molecular biology (Clifton, NJ). Humana. 2024;2786:237–54. 10.1007/978-1-0716-3770-8_11. 10.1007/978-1-0716-3770-8_11 38814398

[11] Zhang H, Zhang L, Lin A, Xu C, Li Z, Liu K, et al. Algorithm for optimized mRNA design improves stability and immunogenicity. Nature. 2023;621:396–403. 10.1038/s41586-023-06127-z. 10.1038/s41586-023-06127-z PMC10499610 37130545

[12] RNA Vaccines. springer us. 2024. 10.1007/978-1-0716-3770-8.

[13] World Health Organization: WHO. WHO.Science Council report on mRNA vaccines [Internet]. 2023. https://cdn.who.int/media/docs/default-source/immunization/mrna-ttp/april-2023/1_adeeba_science_council.pdf?sfvrsn=7b9cf29c_2.

[14] VostrosablinN, Lim S, Gopal P, Brazdilova K, Parajuli S, Wei X, et al. mRNAid, an open-source platform for therapeutic mRNA design and optimization strategies. NAR Genomics and Bioinformatics. Oxford University Press. 2024;6:lqae028. 10.1093/nargab/lqae028. 10.1093/nargab/lqae028 PMC10935487 38482061

[15] Leppek K, Das R, Barna M. Functional 5’ UTR mRNA structures in eukaryotic translation regulation and how to find them. Nat Rev Mol Cell Biol. 2017;19:158–74. 10.1038/nrm.2017.103. 29165424 10.1038/nrm.2017.103 PMC5820134

[16] Shatkin AJ. Capping of eucaryotic mRNAs. Cell. 1976;9:645–53. 10.1016/0092-8674(76)90128-8. 1017010 10.1016/0092-8674(76)90128-8

[17] Kozak M. Point mutations define a sequence flanking the AUG initiator codon that modulates translation by eukaryotic ribosomes. Cell. 1986;44:283–92. 10.1016/0092-8674(86)90762-2. 3943125 10.1016/0092-8674(86)90762-2

[18] Patronov A, Doytchinova I. T-cell epitope vaccine design by immunoinformatics. Open Biology. The Royal Society. 2013;3:120139. 10.1098/rsob.120139. 23303307 10.1098/rsob.120139 PMC3603454

[19] West JD, Smith HJ, Vu LT, Fogarty EA, Matreyek KA, Fowler DM, et al. The quantitative impact of 3’UTRs on gene expression. Nucleic acids research. Oxford University Press. 2025;53. 10.1093/nar/gkaf568. 10.1093/nar/gkaf568 PMC12207410 40586305

[20] Ma X, Liu S, Fan B, Jin D, Miao L, Liu L, et al. Enhancing mRNA translation efficiency by introducing sequence optimized AU-rich elements in 3’ UTR via HuR anchorage. Mol Ther Nucleic Acids. 2025;36:102485. 10.1016/j.omtn.2025.102485. 40125272 10.1016/j.omtn.2025.102485 PMC11930071

[21] Wan Y, Kertesz M, Spitale RC, Segal E, Chang HY. Understanding the transcriptome through RNA structure. Nat Rev Genet. 2011;12:641–55. 10.1038/nrg3049. 21850044 10.1038/nrg3049 PMC3858389

[22] Tanguay RL, Gallie DR. Translational efficiency is regulated by the length of the 3′ untranslated region. Molecular and Cellular Biology. Taylor & Francis. 1996;16:146–56. 10.1128/MCB.16.1.146. 8524291 10.1128/mcb.16.1.146 PMC230988

[23] Zhang J, Kuo CJ, Chen L. GC content around splice sites affects splicing through pre-mRNA secondary structures. BMC Genom. 2011;12:90. 10.1186/1471-2164-12-90. 21281513 10.1186/1471-2164-12-90 PMC3041747

[24] Courel M, Clément Y, Bossevain C, Foretek D, Vidal Cruchez O, Yi Z, et al. GC content shapes mRNA storage and decay in human cells. eLife. Elife Sciences Publications. 2019;8. 10.7554/eLife.49708. 10.7554/eLife.49708 PMC6944446 31855182

[25] Kirshina A, Vasileva O, Kunyk D, Seregina K, Muslimov A, Ivanov R, et al. Effects of combinations of untranslated-region sequences on translation of mRNA. biomolecules. Multidisciplinary Digital Publishing Institute (Mdpi). 2023;13:1677. 10.3390/biom13111677. 10.3390/biom13111677 PMC10669451 38002359

[26] Shepelev NM, Razumova EA, Lavrov AI, Kiniry SJ, Makaryuk AM, Bibisheva RD, et al. Translation of numerous upstream open reading frames rather than their products is essential for proliferation of human cells of distinct origin. bioRxiv (Cold Spring Harbor Laboratory). Royal Society. 2025. 10.1101/2025.07.07.663426.

[27] Ryczek N, Łyś A, Makałowska I. The functional meaning of 5’UTR in protein-coding genes. International journal of molecular sciences. Multidisciplinary Digital Publishing Institute (Mdpi). 2023;24:2976. 10.3390/ijms24032976. 36769304 10.3390/ijms24032976 PMC9917990

[28] Kertesz M, Wan Y, Mazor E, Rinn JL, Nutter RC, Chang HY, et al. Genome-wide measurement of RNA secondary structure in yeast. Nature. 2010;467:103–7. 10.1038/nature09322. 20811459 10.1038/nature09322 PMC3847670

[29] Liu Y, Gao J, Zhang X, Fang X. Joint design of 5’ untranslated region and coding sequence of mRNA. arXiv (Cornell University). TU Dresden. 2024. 10.48550/arXiv.2410.20781.

[30] Mauger DM, Cabral BJ, Presnyak V, Su SV, Reid DW, Goodman B, et al. mRNA structure regulates protein expression through changes in functional half-life. Proceedings of the National Academy of Sciences of the United States of America. Natl Acad Sci. 2019;116:24075–83. 10.1073/pnas.1908052116. 31712433 10.1073/pnas.1908052116 PMC6883848

[31] Ye Z, Bonam SR, Mckay LGA, Plante JA, Walker J, Zhao Y, et al. Monovalent SARS-COV-2 mRNA vaccine using optimal UTRs and LNPs is highly immunogenic and broadly protective against Omicron variants. Proceedings of the National Academy of Sciences of the United States of America. Natl Acad Sci. 2023;120:e2311752120. 10.1073/pnas.2311752120. 10.1073/pnas.2311752120 PMC10756290 38134199

[32] Ding X, Zhou Y, He J, Zhao J, Li J. Enhancement of SARS-CoV-2 mRNA vaccine efficacy through the application of TMSB10 UTR for superior antigen presentation and immune activation. Vaccines. Multidisciplinary Digital Publishing Institute (Mdpi). 2024;12:432. 10.3390/vaccines12040432. 10.3390/vaccines12040432 PMC11053782 38675814

[33] Lui KH, Geisberg JV, Moqtaderi Z, Struhl K. 3’ untranslated regions are modular entities that determine polyadenylation profiles. Molecular and Cellular Biology. Taylor & Francis. 2022;42:e0024422. 10.1128/mcb.00244-22. 10.1128/mcb.00244-22 PMC9476944 35972270

[34] Wang Q, Chen X, Zhang X-O. The Dynamic landscape of 3’-UTR alternative polyadenylation across mouse fetal development and anatomy. Advanced science (Weinheim, Baden-Wurttemberg, Germany). Wiley-Blackwell. 2025;12:e2502443. 10.1002/advs.202502443. 10.1002/advs.202502443 PMC12097126 40126195

[35] Pandey KN. Functional roles of short sequence motifs in the endocytosis of membrane receptors. Frontiers in bioscience (Landmark edition). Imr Press. 2009;14:5339. 10.2741/3599. 10.2741/3599 PMC2751658 19482617

[36] Novotný J, Handschumacher M, Haber E, Bruccoleri RE, Carlson WB, Fanning DW, et al. Antigenic determinants in proteins coincide with surface regions accessible to large probes (antibody domains). Proceedings of the National Academy of Sciences. Natl Acad Sci. 1986;83:226–30. 10.1073/pnas.83.2.226. 10.1073/pnas.83.2.226 PMC322830 2417241

[37] Paul S, Weiskopf D, Angelo MA, Sidney J, Peters B, Sette A. HLA class I alleles are associated with peptide-binding repertoires of different size, affinity, and immunogenicity. J Immunol Amer Assoc Immunologists. 2013;191:5831–9. 10.4049/jimmunol.1302101. 24190657 10.4049/jimmunol.1302101 PMC3872965

[38] Sette A, Sidney J. Nine major HLA class I supertypes account for the vast preponderance of HLA-A and -B polymorphism. Immunogenet. 1999;50:201–12. 10.1007/s002510050594. 10602880 10.1007/s002510050594

[39] Peng C, Shi C, Cao X, Li Y, Liu F, Lu F. Factors influencing recombinant protein secretion efficiency in gram-positive bacteria: signal peptide and beyond. Front Bioeng Biotechnol. 2019;7:139. 10.3389/fbioe.2019.00139. 10.3389/fbioe.2019.00139 PMC6579943 31245367

[40] Futatsumori-Sugai M, Tsumoto K. Signal peptide design for improving recombinant protein secretion in the baculovirus expression vector system. Biochem Biophys Res Commun. 2009;391:931–5. 10.1016/j.bbrc.2009.11.167. 10.1016/j.bbrc.2009.11.167 19962965

[41] Barazesh M, Abbasi M, Mohammadi M, Nasiri MN, Rezaei F, Mohammadi S, et al. Bioinformatics analysis to design a multi-epitope mRNA vaccine against S. agalactiae exploiting pathogenic proteins. Sci Rep. 2024;14:28294. 10.1038/s41598-024-79503-y. 39550419 10.1038/s41598-024-79503-y PMC11569170

[42] Karikó K, Buckstein M, Ni H, Weissman D. Suppression of RNA recognition by toll-like receptors: The impact of nucleoside modification and the evolutionary origin of RNA. Immun. 2005;23:165–75. 10.1016/j.immuni.2005.06.008. 16111635 10.1016/j.immuni.2005.06.008

[43] Kim S, Jeon JH, Kim M, Lee Y, Hwang Y-H, Park M, et al. Innate immune responses against mRNA vaccine promote cellular immunity through IFN-β at the injection site. Nat Commun. 2024;15:7226. 10.1038/s41467-024-51411-9. 10.1038/s41467-024-51411-9 PMC11349762 39191748

[44] Umitaibatin R, Harisna AH, Jauhar MM, Syaifie PH, Arda AG, Nugroho DW, et al. Immunoinformatics study: Multi-epitope based vaccine design from SARS-CoV-2 spike glycoprotein. Vaccines. Multidisciplinary Digital Publishing Institute (Mdpi). 2023;11:399. 10.3390/vaccines11020399. 10.3390/vaccines11020399 PMC9964839 36851275

[45] MahalikP, Tanya M, Pradhan S, Routraya S, Priyadarsini D, Nayak RK. Immunoinformatics approach for the prediction of novel peptide-based epitope vaccine design against leishmania donovani: A computational biology approach. Int J Multidiscip Res. 2023;5:3. 10.36948/ijfmr.2023.v05i03.3671.

[46] Obaidullah AJ, Alanazi MM, Alsaif NA, Albassam H, Almehizia AA, Alqahtani AM, et al. Immunoinformatics-guided design of a multi-epitope vaccine based on the structural proteins of severe acute respiratory syndrome coronavirus 2†. RSC Advances. R Soc Chem. 2021;11:18103–21. 10.1039/D1RA02885E. 35480208 10.1039/d1ra02885e PMC9033181

[47] Krishnakumari V, Rangaraj N, Nagaraj R. Antifungal activities of human beta-defensins HBD-1 to HBD-3 and Their C-Terminal Analogs Phd1 to Phd3. antimicrobial agents and chemotherapy. ASM. 2008;53:256–60. 10.1128/aac.00470-08. 18809937 10.1128/AAC.00470-08 PMC2612179

[48] Mei H-F, Jin X-B, Zhu J-Y, Zeng A-H, Wu Q, Lu X-M, et al. β-defensin 2 as an adjuvant promotes anti-melanoma immune responses and inhibits the growth of implanted murine melanoma in vivo. PLoS ONE. 2012;7:e31328. 10.1371/journal.pone.0031328. 10.1371/journal.pone.0031328 PMC3278441 22348070

[49] Kim J, Yang YL, Jang S-H, Jang Y-S. Human β-defensin 2 plays a regulatory role in innate antiviral immunity and is capable of potentiating the induction of antigen-specific immunity. J Virol. 2018;15:124. 10.1186/s12985-018-1035-2. 30089512 10.1186/s12985-018-1035-2 PMC6083524

[50] Zhou H, Du X, Wang Y, Kong J, Zhang X, Wang W, et al. Antimicrobial peptide A20L: in vitro and in vivo antibacterial and antibiofilm activity against carbapenem-resistant Klebsiella pneumoniae. Microbiology spectrum. ASM. 2024;12:e0397923. 10.1128/spectrum.03979-23. 10.1128/spectrum.03979-23 PMC11302274 38980018

[51] Hanson G, Coller J. Codon optimality, bias and usage in translation and mRNA decay. Nat Rev Mol Cell Biol. 2017;19:20–30. 10.1038/nrm.2017.91. 29018283 10.1038/nrm.2017.91 PMC6594389

[52] Kudla G, Murray AW, Tollervey D, Plotkin JB. Coding-Sequence Determinants of Gene Expression in Escherichia coli. Science. Am Assoc Adv Sci. 2009;324:255–8. 10.1126/science.1170160. 10.1126/science.1170160 PMC3902468 19359587

[53] Ho LLY, Schiess GHA, Miranda P, Weber G, Astakhova K. Pseudouridine and N1-methylpseudouridine as potent nucleotide analogues for RNA therapy and vaccine development. RSC Chemical Biology. R Soc Chem. 2024;5:418–25. 10.1039/D4CB00022F. 38725905 10.1039/d4cb00022f PMC11078203

[54] Morais P, Adachi H, Yu Y-T. The Critical contribution of pseudouridine to mRNA COVID-19 vaccines. Front Cell Dev Biol. 2021;9:789427. 10.3389/fcell.2021.789427. 34805188 10.3389/fcell.2021.789427 PMC8600071

[55] Jin L, Zhou Y, Zhang S, Chen S-J. mRNA vaccine sequence and structure design and optimization: Advances and challenges. J Biol Chem. 2025;301:108015. 10.1016/j.jbc.2024.108015. 39608721 10.1016/j.jbc.2024.108015 PMC11728972

[56] Kuzmin IV, Soto Acosta R, Pruitt L, Wasdin PT, Kedarinath K, Hernandez KR, et al. Comparison of uridine and N1-methylpseudouridine mRNA platforms in development of an Andes virus vaccine. Nat Commun. 2024;15:6421. 10.1038/s41467-024-50774-3. 39080316 10.1038/s41467-024-50774-3 PMC11289437

[57] Spencer PS, Siller E, Anderson JF, Barral JM. Silent substitutions predictably alter translation elongation rates and protein folding efficiencies. J Mol Biol. 2012;422:328–35. 10.1016/j.jmb.2012.06.010. 10.1016/j.jmb.2012.06.010 PMC3576719 22705285

[58] Hamasaki-Katagiri N, Lin BC, Simon J, Hunt RC, Schiller T, Russek-Cohen E, et al. The importance of mRNA structure in determining the pathogenicity of synonymous and non-synonymous mutations in haemophilia. Haemophilia. 2016;23:e8–17. 10.1111/hae.13107. 10.1111/hae.13107 PMC5226872 27933712

[59] Ma Q, Zhang X, Yang J, Li H, Hao Y, Feng X. Optimization of the 5ʹ untranslated region of mRNA vaccines. Sci Rep. 2024;14:19845. 10.1038/s41598-024-70792-x. 39191885 10.1038/s41598-024-70792-x PMC11349747

[60] Chen S-C, Xu C-T, Chang C-F, Chao T-Y, Lin C-C, Fu P-W, et al. Optimization of 5’UTR to evade SARS-CoV-2 Nonstructural protein 1-directed inhibition of protein synthesis in cells. Appl Microbiol Biotechnol. 2023;107:2451–68. 10.1007/s00253-023-12442-2. 36843199 10.1007/s00253-023-12442-2 PMC9968647

[61] Goodswen SJ, Kennedy PJ, Ellis JT. A guide to current methodology and usage of reverse vaccinology towards in silico vaccine discovery. FEMS Microbiology Reviews. Oxford University Press. 2023;47. 10.1093/femsre/fuad004. 10.1093/femsre/fuad004 36806618

[62] Gandvi D, Jyotishi C, Patel M, Gupta R. In silico design of a multi-epitope pan vaccine targeting Schistosoma species. Genomics & Informatics. Korea Genome Organization. 2025;23:21. 10.1186/s44342-025-00053-4. 10.1186/s44342-025-00053-4 PMC12560484 41146254

[63] Qiu J, Wei Y, Shu J, Zheng W, Zhang Y, Xie J, et al. Integrated in-silico design and in vivo validation of multi-epitope vaccines for norovirus. J Virol. 2025;22:166. 10.1186/s12985-025-02796-6. 40426240 10.1186/s12985-025-02796-6 PMC12117790

[64] Mahmoodi S, Amirzakaria JZ, Ghasemian A. In silico design and validation of a novel multi-epitope vaccine candidate against structural proteins of Chikungunya virus using comprehensive immunoinformatics analyses. PLOS ONE. 2023;18:e0285177. 10.1371/journal.pone.0285177. 10.1371/journal.pone.0285177 PMC10162528 37146081

[65] Clemente B, Denis M, Silveira CP, Schiavetti F, Brazzoli M, Stranges D. Straight to the point: targeted mRNA-delivery to immune cells for improved vaccine design. Front Immunol. 2023;14:1294929. 10.3389/fimmu.2023.1294929. 38090568 10.3389/fimmu.2023.1294929 PMC10711611

[66] Kane JF. Effects of rare codon clusters on high-level expression of heterologous proteins in Escherichia coli. Curr Opin Biotechnol. 1995;6:494–500. 10.1016/0958-1669(95)80082-4 7579660 10.1016/0958-1669(95)80082-4

[67] Hou X, Zaks T, Langer R, Dong Y. Lipid nanoparticles for mRNA delivery. Nat Rev Mater. 2021;6:1078–94. 10.1038/s41578-021-00358-0. 34394960 10.1038/s41578-021-00358-0 PMC8353930

[68] Atanasova M, Dimitrov I, Ralchev N, Markovski A, Manoylov I, Bradyanova S, et al. Design, development and immunogenicity study of a multi-epitope vaccine prototype against SARS-CoV-2. Pharmaceuticals. Multidisciplinary Digital Publishing Institute (Mdpi). 2024;17:1498. 10.3390/ph17111498. 10.3390/ph17111498 PMC11597159 39598409

[69] Song X, Li Y, Wu H, Qiu H, Sun Y. T-Cell epitope-based vaccines: A promising strategy for prevention of infectious diseases. Vaccines. Multidisciplinary Digital Publishing Institute (Mdpi). 2024;12:1181. 10.3390/vaccines12101181. 10.3390/vaccines12101181 PMC11511246 39460347

[70] Bukhari SNH, Jain A, Haq E, Mehbodniya A, Webber J. Machine learning techniques for the prediction of B-Cell and T-Cell epitopes as potential vaccine targets with a specific focus on SARS-CoV-2 pathogen: A review. Pathogens (Basel, Switzerland). Multidisciplinary Digital Publishing Institute (Mdpi). 2022;11:146. 10.3390/pathogens11020146. 10.3390/pathogens11020146 PMC8879824 35215090

[71] Yin L, Stern LJ. Measurement of peptide binding to MHC class II molecules by fluorescence polarization. Curr Protoc Immunol. 2014;106:5.10.1-5.10.12. 10.1002/0471142735.im0510s106. 10.1002/0471142735.im0510s106 PMC4151172 25081912

[72] Sette A, Vitiello A, Reherman B, Fowler P, Nayersina R, Kast WM, et al. The relationship between class I binding affinity and immunogenicity of potential cytotoxic T cell epitopes. J Immunol. 1994;153:5586–92. 10.4049/jimmunol.153.12.5586. 7527444

[73] Reardon B, Koşaloğlu-Yalçın Z, Paul S, Peters B, Sette A. Allele-specific thresholds of eluted ligands for T-Cell epitope prediction. Molecular & Cellular Proteomics: MCP. 2021;20:100122. 10.1016/j.mcpro.2021.100122. 10.1016/j.mcpro.2021.100122 PMC8724920 34303001

[74] Vita R, Mahajan S, Overton JA, Dhanda SK, Martini S, Cantrell JR, et al. The Immune Epitope Database (IEDB): 2018 update. Nucleic Acids Research. Oxford University Press. 2018;47:D339–43. 10.1093/nar/gky1006. 10.1093/nar/gky1006 PMC6324067 30357391

[75] Khan A, Khan S, Saleem S, Nizam-Uddin N, Mohammad A, Khan T, et al. Immunogenomics guided design of immunomodulatory multi-epitope subunit vaccine against the SARS-CoV-2 new variants, and its validation through in silico cloning and immune simulation. Comput Biol Med. 2021;133:104420. 10.1016/j.compbiomed.2021.104420. 33930764 10.1016/j.compbiomed.2021.104420 PMC8064902

[76] Alnajran H, Awadalla M, Aldakheel FM, Alam I, Momin AA, Alturaiki W, et al. Design of a peptide-based vaccine against human respiratory syncytial virus using a reverse vaccinology approach: evaluation of immunogenicity, antigenicity, allergenicity, and toxicity. Front Immunol. 2025;16:1546254. 10.3389/fimmu.2025.1546254. 10.3389/fimmu.2025.1546254 PMC11986473 40226615

[77] Momajadi L, Khanahmad H, Mahnam K. Designing a multi-epitope influenza vaccine: an immunoinformatics approach. Sci Rep. 2024;14:25382. 10.1038/s41598-024-74438-w. 10.1038/s41598-024-74438-w PMC11512060 39455641

[78] Puigbò P, Bravo IG, Garcia-Vallve S. CAIcal: A combined set of tools to assess codon usage adaptation. Biol Direct. 2008;3:38. 10.1186/1745-6150-3-38. 18796141 10.1186/1745-6150-3-38 PMC2553769

[79] Naveed M, Toheed M, Aziz T, Asim M, Qadir P, Rehman HM, et al. Rational computational design and development of an immunogenic multiepitope vaccine incorporating transmembrane proteins of Fusobacterium necrophorum. Sci Rep. 2025;15:15587. 10.1038/s41598-025-00166-4. 40320394 10.1038/s41598-025-00166-4 PMC12050319

[80] Wang Q, Zhang L, Kuwahara K, Li L, Liu Z, Li T, et al. Immunodominant SARS coronavirus epitopes in humans elicited both enhancing and neutralizing effects on infection in non-human primates. ACS infectious diseases. Am Chem Soc. 2016;2:361–76. 10.1021/acsinfecdis.6b00006. 10.1021/acsinfecdis.6b00006 PMC7075522 27627203

[81] Li D-D, Li Q-H. SARS-CoV-2: vaccines in the pandemic era. Mil Med Res. 2021;8:1. 10.1186/s40779-020-00296-y. 33402220 10.1186/s40779-020-00296-y PMC7785400

[82] Amirabadi FSM, Damirchi M, Amani M, Shamami MK, Salehi N, Nafian F. In silico design and evaluation of a multi-epitope vaccine targeting SARS-CoV-2 structural proteins. Curr Proteom. 2026;23:100088. 10.1016/j.curpro.2026.100088.

[83] Rehman A, Ahmad S, Shahid F, Albutti A, Alwashmi ASS, Aljasir MA, et al. Integrated core proteomics, subtractive proteomics, and immunoinformatics investigation to unveil a potential multi-epitope vaccine against schistosomiasis. vaccines. Multidisciplinary Digital Publishing Institute (Mdpi). 2021;9:658. 10.3390/vaccines9060658. 10.3390/vaccines9060658 PMC8235758 34208663

[84] Alshabrmi FM, Alatawi EA. Subtractive proteomics-guided vaccine targets identification and designing of multi-epitopes vaccine for immune response instigation against Burkholderia pseudomallei. Int J Biol Macromol. 2024;270:132105. 10.1016/j.ijbiomac.2024.132105. 10.1016/j.ijbiomac.2024.132105 38710251

[85] Pucci F, Rooman M. Prediction and evolution of the molecular fitness of SARS-CoV-2 variants: Introducing SpikePro. Viruses. Multidisciplinary Digital Publishing Institute (Mdpi). 2021;13:935. 10.3390/v13050935. 34070055 10.3390/v13050935 PMC8158131

[86] Chaudhary N, Weissman D, Whitehead KA. mRNA vaccines for infectious diseases: principles, delivery and clinical translation. Nat Rev Drug Discov. 2021;20:817–38. 10.1038/s41573-021-00283-5. 34433919 10.1038/s41573-021-00283-5 PMC8386155

[87] Polack FP, Thomas SJ, Kitchin N, Absalon J, Gurtman A, Lockhart S, et al. Safety and efficacy of the BNT162b2 mRNA Covid-19 vaccine. N Engl J Med. 2020;383:2603–15. 10.1056/NEJMoa2034577. 33301246 10.1056/NEJMoa2034577 PMC7745181

[88] Walls AC, Park Y-J, Tortorici MA, Wall A, Mcguire AT, Veesler D. Structure, function, and antigenicity of the SARS-CoV-2 Spike Glycoprotein. Cell. 2020;181:281–292.e6. 10.1016/j.cell.2020.02.058. 32155444 10.1016/j.cell.2020.02.058 PMC7102599

[89] Ke Z, Oton J, Qu K, Cortese M, Zila V, Mckeane L, et al. Structures and distributions of SARS-CoV-2 spike proteins on intact virions. Nature. 2020;588:498–502. 10.1038/s41586-020-2665-2. 32805734 10.1038/s41586-020-2665-2 PMC7116492

[90] Wrapp D, Wang N, Corbett KS, Goldsmith JA, Hsieh C-L, Abiona O, et al. Cryo-EM structure of the 2019-nCoV spike in the prefusion conformation. Science. 2020;367:1260–3. 10.1126/science.abb2507. 10.1126/science.abb2507 PMC7164637 32075877

[91] Duan L, Zheng Q, Zhang H, Niu Y, Lou Y, Wang H. The SARS-CoV-2 Spike Glycoprotein biosynthesis, structure, function, and antigenicity: Implications for the design of spike-based vaccine immunogens. Front Immunol. 2020;11:576622. 10.3389/fimmu.2020.576622. 33117378 10.3389/fimmu.2020.576622 PMC7575906

[92] Grifoni A, Weiskopf D, Ramirez SI, Mateus J, Dan JM, Moderbacher CR, et al. Targets of T Cell responses to SARS-CoV-2 coronavirus in humans with COVID-19 disease and unexposed individuals. Cell. 2020;181:1489–1501.e15. 10.1016/j.cell.2020.05.015. 32473127 10.1016/j.cell.2020.05.015 PMC7237901

[93] Shomuradova AS, Vagida MS, Sheetikov SA, Zornikova KV, Kiryukhin D, Titov A, et al. SARS-CoV-2 epitopes are recognized by a public and diverse repertoire of human T cell receptors. Immun. 2020;53:1245–1257.e5. 10.1016/j.immuni.2020.11.004. 33326767 10.1016/j.immuni.2020.11.004 PMC7664363

[94] Federico L, Malone B, Tennøe S, Chaban V, Osen JR, Gainullin M, et al. Experimental validation of immunogenic SARS-CoV-2 T cell epitopes identified by artificial intelligence. Front Immunol. 2023;14:1265044. 10.3389/fimmu.2023.1265044. 38045681 10.3389/fimmu.2023.1265044 PMC10691274

[95] Mateus J, Grifoni A, Tarke A, Sidney J, Ramirez SI, Dan JM, et al. Selective and cross-reactive SARS-CoV-2 T cell epitopes in unexposed humans. Science. 2020;370:89–94. 10.1126/science.abd3871. 10.1126/science.abd3871 PMC7574914 32753554

[96] Sholukh AM, Fiore-Gartland A, Ford ES, Miner MD, Hou YJ, Tse LV, et al. Evaluation of cell-based and surrogate SARS-CoV-2 neutralization assays. J Clin Microbiol. 2021;59:e0052721. 10.1128/JCM.00527-21. 10.1128/JCM.00527-21 PMC8451402 34288726

[97] Peng Y, Mentzer AJ, Ansari A, Liu G, Yao X, Yin Z, et al. Broad and strong memory CD4+ and CD8+ T cells induced by SARS-CoV-2 in UK convalescent individuals following COVID-19. Nat Immunol. 2020;21:1336–45. 10.1038/s41590-020-0782-6. 32887977 10.1038/s41590-020-0782-6 PMC7611020

[98] Karsten H, Cords L, Westphal T, Knapp M, Brehm TT, Hermanussen L, et al. High-resolution analysis of individual spike peptide-specific CD4+ T-cell responses in vaccine recipients and COVID-19 patients. Clin Transl Immunol. 2022;11:e1410. 10.1002/cti2.1410. 10.1002/cti2.1410 PMC9363231 35957961

[99] Sohail MS, Ahmed SF, Quadeer AA, Mckay MR. In silico T cell epitope identification for SARS-CoV-2: Progress and perspectives. Adv Drug Deliv Rev. 2021;171:29–47. 10.1016/j.addr.2021.01.007. 33465451 10.1016/j.addr.2021.01.007 PMC7832442

[100] Li L, Zhao Z, Yang X, Li W, Chen S, Sun T, et al. Identification of four linear B-cell epitopes on the SARS-CoV-2 spike protein able to elicit neutralizing antibodies. bioRxiv. 2020. 10.1101/2020.12.13.422550.

[101] Jumper J, Evans R, Pritzel A, Green T, Figurnov M, Ronneberger O, et al. Highly accurate protein structure prediction with AlphaFold. Nature. 2021;596:583–9. 10.1038/s41586-021-03819-2. 34265844 10.1038/s41586-021-03819-2 PMC8371605

[102] Volkan E. COVID-19: Structural considerations for virus pathogenesis, therapeutic strategies and vaccine design in the novel SARS-CoV-2 variants era. Mol Biotechnol. 2021;63:885–97. 10.1007/s12033-021-00353-4. 34145550 10.1007/s12033-021-00353-4 PMC8213040

[103] Tahir Ul Qamar M, Rehman A, Tusleem K, Ashfaq UA, Qasim M, Zhu X, et al. Designing of a next generation multiepitope based vaccine (MEV) against SARS-COV-2: Immunoinformatics and in silico approaches. PLOS ONE. 2020;15:e0244176. 10.1371/journal.pone.0244176. 10.1371/journal.pone.0244176 PMC7755200 33351863

[104] Srinivasan S, Selvaraj GF, Gopalan V, Padmanabhan P, Ramesh K, Govindan K, et al. Epitope identification and designing a potent multi-epitope vaccine construct against SARS-CoV-2 including the emerging variants. J Glob Infect Dis. 2022;14:24–30. 10.4103/jgid.jgid_96_21. 35418729 10.4103/jgid.jgid_96_21 PMC8996455

[105] Bui H-H, Sidney J, Dinh K, Southwood S, Newman MJ, Sette A. Predicting population coverage of T-cell epitope-based diagnostics and vaccines. BMC Bioinfo. 2006;7:153. 10.1186/1471-2105-7-153. 10.1186/1471-2105-7-153 PMC1513259 16545123

[106] Dong R, Chu Z, Yu F, Zha Y. Contriving Multi-Epitope Subunit of Vaccine for COVID-19: Immunoinformatics Approaches. Front Immunol. 2020;11:1784. 10.3389/fimmu.2020.01784. 32849643 10.3389/fimmu.2020.01784 PMC7399176

[107] Khairkhah N, Bolhassani A, Agi E, Namvar A, Nikyar A. Immunological investigation of a multiepitope peptide vaccine candidate based on main proteins of SARS-CoV-2 pathogen. PLoS ONE. 2022;17:e0268251. 10.1371/journal.pone.0268251. 10.1371/journal.pone.0268251 PMC9182696 35679246

[108] Menzella HG. Comparison of two codon optimization strategies to enhance recombinant protein production in Escherichia coli. Microb Cell Fact. 2011;10:15. 10.1186/1475-2859-10-15. 21371320 10.1186/1475-2859-10-15 PMC3056764

[109] Wu X, Shan K, Zan F, Tang X, Qian Z, Lu J. Optimization and deoptimization of codons in SARS‐CoV‐2 and related implications for vaccine development. Adv Sci. 2023;10:e2205445. 10.1002/advs.202205445. 10.1002/advs.202205445 PMC10427376 37267926

[110] Li Y, Wang F, Yang J, Han Z, Chen L, Jiang W, et al. Deep generative optimization of mRNA codon sequences for enhanced mRNA translation and therapeutic efficacy. Nat Commun. 2025;16:9957. 10.1038/s41467-025-64894-x. 41224770 10.1038/s41467-025-64894-x PMC12612108

[111] Babendure JR, Babendure JL, Ding J-H, Tsien RY. Control of mammalian translation by mRNA structure near caps. RNA. Cold Spring Harbor Laboratory Press. 2006;12:851–61. 10.1261/rna.2309906. 16540693 10.1261/rna.2309906 PMC1440912

[112] Maertens B, Spriestersbach A, Von Groll U, Roth U, Kubicek J, Gerrits M, et al. Gene optimization mechanisms: A multi‐gene study reveals a high success rate of full‐length human proteins expressed in Escherichia coli. Protein Sci. 2010;19:1312–26. 10.1002/pro.408. 20506237 10.1002/pro.408 PMC2970903

[113] Araf Y, Moin AT, Timofeev VI, Faruqui NA, Saiara SA, Ahmed N, et al. Immunoinformatic design of a multivalent peptide vaccine against mucormycosis: Targeting FTR1 protein of major causative fungi. Front Immunol. 2022;13:863234. 10.3389/fimmu.2022.863234. 10.3389/fimmu.2022.863234 PMC9204303 35720422

[114] Tarke A, Grifoni A, Sette A. Bioinformatic and experimental analysis of T Cell immune reactivity to SARS-CoV-2 and its variants. Front Bioinform. 2022;2:876380. 10.3389/fbinf.2022.876380. 36304267 10.3389/fbinf.2022.876380 PMC9580847

