# Supplementary material for: A protocol for computational design of mRNA vaccines with high functionality and specificity
Source: Biol Direct. 2026 Jun 13;21:103. doi: 10.1186/s13062-026-00846-9 (PMC13270589; doi:10.1186/s13062-026-00846-9)
Supplement: Supplementary file 1 — Supplementary material 1 [file 13062_2026_846_MOESM1_ESM.docx]

**Supplementary table S1:** Predicted and shortlisted T-cell epitopes, including MHC class I (CTL), MHC class II (HTL), and B-cell epitopes, with their IEDB peptide reference IDs, residue positions, predicted scores, and percentile ranks. The corresponding HLA alleles, along with allergenicity, toxicity, and antigenicity indices, are also reported.

| **MHC Class I (CTL) Epitopes** | | | | | | | | | | | | |
| --- | --- | --- | --- | --- | --- | --- | --- | --- | --- | --- | --- | --- |
| **Peptide Sequence** | **IEDB Peptide Reference ID** | **Peptide Position (Start-End)** | **HLA Molecules (Strong Binding)** | **Percentile Rank** | **Antigenicity Score** | | **Antigenicity** | | | | **Allergenicity** | **Toxicity** |
| YLQPRTFLL | 1309147 | 269-277 | HLA-A*02:01, HLA-A*02:03, HLA-A*02:06, HLA-A*24:02, HLA-A*32:01, HLA-A*23:01, HLA-B*08:01 | 0.02 | 0.45 | | Antigenic | | | | Non-Allergenic | Non-Toxin |
| LLFNKVTLA | 37289 | 821-829 | HLA-A*02:01, HLA-A*02:03, HLA-A*02:06 | 0.03 | 0.61 | | Antigenic | | | | Non-Allergenic | Non-Toxin |
| IAIVMVTIM | 2249088 | 1225-1233 | HLA-B*35:01, HLA-B*51:01 | 0.57 | 1.13 | | Antigenic | | | | Non-Allergenic | Non-Toxin |
| WTAGAAAYY | 1327824 | 258-266 | HLA-A*01:01, HLA-A*26:01, HLA-A*30:02, HLA-A*68:01, HLA-B*15:01, HLA-B*35:01 | 0.03 | 0.63 | | Antigenic | | | | Non-Allergenic | Non-Toxin |
| SPRRARSVA | 1311590 | 680-688 | HLA-B*07:02 | 0.04 | 0.77 | | Antigenic | | | | Non-Allergenic | Non-Toxin |
| **MHC Class II (HTL) Epitopes** | | | | | | | | | | | | |
| **Peptide Sequence** | **IEDB Peptide Reference ID** | **Peptide Position (Start-End)** | **HLA Molecules** | **Percentile Rank** | **Antigenicity Score** | | **Antigenicity** | | | | **Allergenicity** | **Toxicity** |
| CTFEYVSQPFLMDLE | 1309110 | 166-180 | HLA-DPA1*01:03, HLA-DPA1*02:01, HLA-DPA1*03:01, HLA-DPB1*01:01, HLA-DPB1*04:01, HLA-DPB1*04:02, HLA-DPB1*05:01 | 0.14 | 0.57 | | Antigenic | | | | Non-Allergenic | Non-Toxin |
| VVLSFELLHAPATVC | 1073956 | 511-525 | HLA-DRB1*01:01 | 1.4 | 0.86 | | Antigenic | | | | Non-Allergenic | Non-Toxin |
| GIYQTSNFRVQPTES | 1310445 | 311-325 | HLA-DPA1*01:03, HLA-DPB1*02:01 | 0.79 | 0.86 | | Antigenic | | | | Non-Allergenic | Non-Toxin |
| RFASVYAWNRKRISN | 1310765 | 346-360 | HLA-DRB1*13:02 | 0.97 | 0.42 | | Antigenic | | | | Non-Allergenic | Non-Toxin |
| FKIYSKHTPINLVRD | 1310401 | 201-215 | HLA-DRB1*07:01 | 1.6 | 0.72 | | Antigenic | | | | Non-Allergenic | Non-Toxin |
| **Linear B cell Epitopes** | | | | | | | | | | |  |  |
| Peptide Sequence | IEDB Peptide Reference ID | Peptide Position (Start-End) | Antigenicity_Score | Antigenicity | | Allergenicity | | | Toxicity | |  |  |
| YNSASFSTFKCYGVSPTKLNDLCFT | 1594118 | 369-393 | 1.4031 | Antigenic | | Non-Allergenic | | | Non-Toxin | |  |  |
| GDEVRQIAPGQTGKIADYNYKLP | 1571473 | 404-426 | 1.1017 | Antigenic | | Non-Allergenic | | | Non-Toxin | |  |  |
| YQPYRVVVLSFELLH | 1314034 | 505-519 | 0.9711 | Antigenic | | Non-Allergenic | | | Non-Toxin | |  |  |
| APGQTGKIADYNYKLPDDFT | 2237397 | 346-360 | 1.0425 | Antigenic | | Non-Allergenic | | | Non-Toxin | |  |  |
| CVNFNFNGLTGTGVL | 7289 | 538-552 | 1.3281 | Antigenic | | Non-Allergenic | | | Non-Toxin | |  |  |
| **Confirmational B cell Epitopes** | | | | | | | | | | | | |
| Residues | | | | | | | | Number of residues | | Score | | |
| A:A27, A:Y28, A:T29, A:N30, A:S31, A:F32, A:T33, A:R34, A:G35, A:V36, A:Y37, A:Y38, A:K41, A:L56, A:P57, A:F58, A:F59, A:S60, A:N61, A:V62, A:T63, A:W64, A:F65, A:H66, A:A67, A:I68, A:H69, A:D80, A:N81, A:P82, A:V83, A:L84, A:P85, A:F86, A:N87, A:D88, A:G89, A:V90, A:Y91, A:F92, A:A93, A:S94, A:T95, A:E96, A:K97, A:S98, A:N99, A:I100, A:I101, A:R102, A:G103, A:W104, A:I105, A:F106, A:G107, A:T108, A:T109, A:L110, A:D111, A:S112, A:K113, A:T114, A:Q115, A:S116, A:L117, A:L118, A:I119, A:V120, A:N121, A:N122, A:A123, A:T124, A:N125, A:V126, A:V127, A:I128, A:K129, A:V130, A:C131, A:E132, A:F133, A:Q134, A:F135, A:C136, A:N137, A:D138, A:P139, A:F140, A:L141, A:G142, A:V143, A:N165, A:C166, A:T167, A:F168, A:E169, A:Y170, A:V171, A:S172, A:F186, A:K187, A:N188, A:L189, A:R190, A:E191, A:F192, A:V193, A:F194, A:K195, A:N196, A:D198, A:G199, A:F201, A:K202, A:I203, A:Y204, A:S205, A:K206, A:H207, A:T208, A:P209, A:I210, A:N211, A:L212, A:V213, A:R214, A:D215, A:L216, A:P217, A:Q218, A:G219, A:F220, A:S221, A:A222, A:L223, A:E224, A:P225, A:L226, A:V227, A:D228, A:L229, A:P230, A:I231, A:G232, A:I233, A:N234, A:I235, A:T236, A:R237, A:F238, A:Q239, A:T240, A:L241, A:L242, A:A243, A:L244, A:H245, A:A263, A:A264, A:Y265, A:Y266, A:V267, A:G268, A:Y269, A:L270, A:N280, A:E281, A:N282, A:G283, A:T284, A:T286, C:I326, C:V327, C:R328, C:F329, C:P330, C:N331, C:I332, C:T333, C:N334, C:L335, C:C336, C:P337, C:F338, C:G339, C:E340, C:V341, C:F342, C:N343, C:A344, C:T345, C:R346, C:F347, C:A348, C:S349, C:V350, C:Y351, C:A352, C:W353, C:N354, C:R355, C:K356, C:R357, C:I358, C:S359, C:N360, C:C361, C:V362, C:A363, C:D364, C:S366, C:V367, C:L368, C:N370, C:S371, C:A372, C:S373, C:F374, C:N388, C:D389, C:C391, C:F392, C:T393, C:Y396, C:A397, C:S399, C:F400, C:V401, C:I402, C:R403, C:G404, C:I418, C:N422, C:Y423, C:W436, C:N437, C:S438, C:N439, C:N440, C:L441, C:D442, C:S443, C:K444, C:G447, C:N448, C:Y449, C:N450, C:Y451, C:L452, C:Y453, C:R454, C:R466, C:D467, C:I468, C:F490, C:P491, C:L492, C:Q493, C:S494, C:Y495, C:G496, C:F497, C:Q498, C:P499, C:T500, C:N501, C:V503, C:G504, C:Y505, C:Q506, C:P507, C:Y508, C:R509, C:P521, C:A522, C:T523, C:V524, C:C525, C:G526, C:P527, C:K528, C:K529, C:S530, C:T531, C:N532, C:L533, C:V534, C:K535, C:N536, C:K537, C:N544, C:T553, C:E554, C:S555, C:N556, C:K557, C:K558, C:F559, C:L560, C:P561, C:F562, C:Q563, C:Q564, C:R577, C:D578, C:P579, C:Q580, C:T581, C:L582, C:E583, C:I584, C:L585 | | | | | | | | 312 | | 0.744 | | |
| A:N616, A:C617, A:T618, A:E619, A:N641, A:V642, A:G652, A:A653, A:E654, A:H655, A:V656, A:N657, A:N658, A:S659, A:Y660, A:Y674, A:Q675, A:T676, A:S689, A:Q690, A:S691, A:I693, A:S698 | | | | | | | | 23 | | 0.578 | | |
| B:N616, B:C617, B:T618, B:E619, B:N641, B:V642, B:G652, B:A653, B:E654, B:H655, B:V656, B:N657, B:N658, B:S659, B:Y660, B:Y674, B:Q675, B:T676, B:S689, B:Q690, B:S691, B:I693, B:S698 | | | | | | | | 23 | | 0.578 | | |
| C:N616, C:C617, C:T618, C:E619, C:N641, C:V642, C:G652, C:A653, C:E654, C:H655, C:V656, C:N657, C:N658, C:S659, C:Y660, C:Y674, C:Q675, C:T676, C:S689, C:Q690, C:S691, C:I693, C:S698 | | | | | | | | 23 | | 0.578 | | |
