# Supplementary material for: A protocol for computational design of mRNA vaccines with high functionality and specificity
Source: Biol Direct. 2026 Jun 13;21:103. doi: 10.1186/s13062-026-00846-9 (PMC13270589; doi:10.1186/s13062-026-00846-9)
Supplement: Supplementary file 2 — Supplementary material 2 [file 13062_2026_846_MOESM2_ESM.docx]

**Supplementary table S2:** A list of common Tools and packages used in the procedure of mRNA vaccine design.

| Tool | Description | Latest Version | Reference |
| --- | --- | --- | --- |
| UniProt | A database for exploring protein sequences with detailed information about their structure and function. | Continuously updated [Release 2024_06] | [1] |
| GenBank | A repository for genetic sequences, offering essential data for understanding organisms, including viral genomes. | Continuously updated [Release 263.0] | [2] |
| Prokka | Genome annotation tool for prokaryotic sequences. | v1.14 or later | [3] |
| BlAST | Tool for sequence alignment and validation. | v2.13.0 or later | [4] |
| Pfam | Tool identifies and classifies protein families and domains. | 37.1 | [5] |
| MEME Suite | Tool finds recurring patterns or motifs in sequences. | 5.5.7 | [6] |
| PDB | Repository for experimentally determined 3D structures of biological macromolecules. | Current release (accessed April 2026) | [7] |
| NetMHCpan | Tool predicts which parts of a protein can bind to MHC molecules, helping assess their potential as T-cell epitopes. | 4.1 | [8] |
| AlphaFold | Predicts 3D structure of viral proteins based on sequence. | v2.3.1 | [9] |
| UTRdb | A database used to retrieve and annotate 5 &3 UTRs. | v2.0 updated 2002 | [10] |
| RNAfold | Tool predicts the structure of RNA molecules and calculates their stability. | 2.5.1 | [11] |
| RNAplfold | Tool predicts local RNA secondary structure and estimates nucleotide accessibility for off-target binding. | ViennaRNA Package, v2.7.0 | [11] |
| LinearDesign | Tool optimizes mRNA sequences by balancing codon usage and structural stability. | First version | [12] |
| RNAeval | Tool for calculating the free energy of a given RNA secondary structure using thermodynamic decomposition and analyzing RNA structure to determine how accessible regions are for interactions. | Part of ViennaRNA Suite 2.6.3 | [11] |
| IEDB Analysis Resource | Immunological database for epitope prediction. | v3.0 | [13] |
| Nextflow / Snakemake | Workflow management system for scalable computation. | Nextflow 24.10.2, Snakemake 8.25.5 | [14]  [15] |
| Vaxijen | Tool for antigenicity prediction without sequence alignment. | V2.0 | [16] |
| Allertop | Tool for allergenicity prediction using machine learning. | V2.0 | [17] |
| ToxinPred | Tool for *in silico* toxicity prediction of peptide sequences. | V1.0 | [18] |
| Reverse Translate | Converts protein sequences to nucleotide sequences using codon tables. | Part of SMS2 suite (v1.0) | [19] |
| Expasy Translate | Translate is a tool which allows the translation of a nucleotide (DNA/RNA) sequence to a protein sequence. | SIB Swiss Institute of Bioinformatics (accessed on 2026) | [20] |
| Biomodel | Transcription and Translation Tool. Converts sequences from DNA to RNA and from this to protein. | BiModel Web Server (2020) | [21] |
| ClustalO | Tool for multiple sequence alignment and generation of consensus or conserved sequences. | Clustal Omega (v1.2.4, 2022) | [22] |
| Addgene | Nonprofit plasmid repository for sharing and accessing curated DNA constructs and vectors. | Current release (2026) | [23] |
| NovoPro ExpOptimizer | Tool for codon optimization and reverse translation to enhance gene expression in a selected host organism. | NovoPro ExpOptimizer (web-based tool, accessed 2026) | [24] |
| SnapGene | Tool for molecular cloning simulation, plasmid visualization, and *in silico* validation of DNA constructs. | SnapGene (v6.0, 2023) | [25] |
| C-ImmSim | Tool for *in silico* simulation of immune responses to predict antigenicity and immune system dynamics. | C-ImmSim server (v10.1, 2023) | [26] |
| ProtParam | Tool for computation of physicochemical properties of proteins including molecular weight, pI, stability, and hydropathicity. | ExPASy ProtParam (web-based tool, accessed 2026) | [27] |
| ClusPro | Automated protein–protein docking server to predict the most probable binding conformations and interaction complexes between biomolecules. | ClusPro, Boston University (web server, accessed on 2026) | [28] |
| PyMOL | Molecular visualization tool for three-dimensional rendering, structural analysis, and graphical representation of protein structures. | PyMOL Molecular Graphics System (V2.5, Schrödinger, LLC). | [29] |
| GROMACS | High-performance molecular dynamics simulation package to study the structural dynamics, stability, and interactions of biomolecular systems. | Version 2021.4 | [30] |
| PSIPRED | Neural network-based tool for prediction of protein secondary structure using position-specific scoring matrices derived from sequence alignments. | PSIPRED Workbench, (V4.0, University College London) | [31] |

# References

[1] T. U. Consortium, “UniProt: the universal protein knowledgebase,” *(2016). Nucleic Acids Research*, 2016, doi: 10.1093/nar/gkw1099.

[2] D. A. Benson, M. Cavanaugh, K. Clark, I. Karsch-Mizrachi, D. J. Lipman, and J. Ostell, “GenBank,” *Nucleic Acids Res.*, 2013, doi: 10.1093/nar/gks1195.

[3] T. Seemann, “Prokka: Rapid prokaryotic genome annotation,” *Bioinformatics*, vol. 30, no. 14, pp. 2068–2069, 2014, doi: 10.1093/bioinformatics/btu153.

[4] C. Camacho, G. Coulouris, V. Avagyan, N. Ma, J. Papadopoulos, and K. Bealer, “BLAST+: Architecture and applications,” *BMC Bioinformatics*, vol. 10, no. 1, pp. 1471–2105, 2009, doi: 10.1186/1471-2105-10-421.

[5] A. Bateman, “The PFAM Protein Families database,” *Nucleic Acids Res.*, vol. 30, no. 1, pp. 276–280, 2002, doi: 10.1093/nar/30.1.276.

[6] T. L. Bailey, M. Boden, F. A. Buske, M. Frith, C. E. Grant, and L. Clementi, “MEME Suite: Tools for motif discovery and searching,” *Nucleic Acids Res.*, 2009, doi: 10.1093/nar/gkp335.

[7] H. M. Berman *et al.*, “The Protein Data Bank,” *Nucleic Acids Res.*, vol. 28, no. 1, pp. 235–242, 2000, doi: 10.1093/nar/28.1.235.

[8] V. Jurtz, S. Paul, M. Andreatta, P. Marcatili, and B. Peters, “NetMHCpan-4.1: Improved predictions of MHC antigen presentation by concurrent motif deconvolution and integration of MS MHC eluted ligand data,” *Nucleic Acids Res.*, 2017, doi: 10.1093/nar/gkaa379.

[9] J. Jumper, R. Evans, A. Pritzel, T. Green, M. Figurnov, and O. Ronneberger, “Highly accurate protein structure prediction with AlphaFold,” *Nature*, vol. 596, no. 7873, pp. 583–589, 2021, doi: 10.1038/s41586-021-03819-2.

[10] G. Pesole, “UTRdb and UTRsite: specialized databases of sequences and functional elements of 5’ and 3’ untranslated regions of eukaryotic mRNAs,” *Update 2002. Nucleic Acids Research*, vol. 30, no. 1, pp. 335–340, 2002, doi: 10.1093/nar/30.1.335.

[11] R. Lorenz *et al.*, “ViennaRNA Package 2.0,” *Algorithms for Molecular Biology*, vol. 6, pp. 1748–7188, 2011, doi: 10.1186/1748-7188-6-26.

[12] H. Zhang *et al.*, “Algorithm for optimized mRNA design improves stability and immunogenicity,” *Nature*, vol. 621, no. 7978, pp. 396–403, 2023, doi: 10.1038/s41586-023-06127-z.

[13] R. Vita, S. Mahajan, J. A. Overton, S. K. Dhanda, S. Martini, and J. R. Cantrell, “The Immune Epitope Database (IEDB): 2018 update,” *Nucleic Acids Res.*, 2019, doi: 10.1093/nar/gky1006.

[14] J. Köster and S. Rahmann, “Snakemake—a scalable bioinformatics workflow engine,” *Bioinformatics*, vol. 28, no. 19, pp. 2520–2522, 2012, doi: 10.1093/bioinformatics/bts480.

[15] P. Di Tommaso, M. Chatzou, E. W. Floden, P. P. Barja, and E. Palumbo, “Nextflow enables reproducible computational workflows,” *Nat. Biotechnol.*, vol. 35, no. 4, pp. 316–319, 2017, doi: 10.1038/nbt.3820.

[16] I. A. Doytchinova and D. R. Flower, “VaxiJen: a server for prediction of protective antigens, tumour antigens and subunit vaccines,” *BMC Bioinformatics*, vol. 8, pp. 1471–2105, 2007, doi: 10.1186/1471-2105-8-4.

[17] I. Dimitrov, I. Bangov, D. R. Flower, and I. Doytchinova, “AllerTOP v.2—a server for in silico prediction of allergens,” *J. Mol. Model.*, vol. 20, pp. 814–894, 2014, doi: 10.1007/s00894-014-2278-5.

[18] S. Gupta *et al.*, “In silico approach for predicting toxicity of peptides and proteins,” *PLoS One*, vol. 8, no. 9, p. e73957, 2013, doi: 10.1371/journal.pone.0073957.

[19] P. Stothard, “The Sequence Manipulation Suite: JavaScript programs for analyzing and formatting protein and DNA sequences,” *Nucleic Acids Res.*, vol. 28, no. 1, pp. 41–42, 2000, doi: 10.1093/nar/28.1.41.

[20] S. Duvaud, C. Gabella, F. Lisacek, H. Stockinger, V. Ioannidis, and C. Durinx, “ExPASy, the Swiss Bioinformatics Resource Portal, as designed by its users,” *Nucleic Acids Res.*, vol. 49, no. W1, pp. W216–W227, 2021, doi: 10.1093/nar/gkab225.

[21] J. Singh, J. Hanson, K. Paliwal, and Y. Zhou, “RNA secondary structure prediction using an ensemble of two-dimensional deep neural networks and transfer learning,” *Bioinformatics*, vol. 35, no. 14, pp. 2345–2352, 2019, doi: 10.1093/bioinformatics/btz085.

[22] F. Sievers and D. G. Higgins, “Clustal Omega for making accurate alignments of many protein sequences,” *Protein Science*, vol. 27, no. 1, pp. 135–145, 2018, doi: 10.1002/pro.3290.

[23] J. Kamens, “The Addgene repository: an international nonprofit plasmid and data resource,” *Nucleic Acids Res.*, vol. 43, no. D1, pp. D1152–D1157, 2015, doi: 10.1093/nar/gku893.

[24] NovoPro Bioscience, “NovoPro Codon Optimization Tool,” *Online Resource*, 2026, [Online]. Available: https://www.novoprolabs.com/tools/codon-optimization

[25] GraphPad Software LLC, “SnapGene: Software for Everyday Molecular Biology,” *Online Resource*, 2026, [Online]. Available: https://www.snapgene.com/

[26] N. Rapin, O. Lund, M. Bernaschi, and F. Castiglione, “Computational immunology meets bioinformatics: The use of prediction tools for molecular binding in the simulation of the immune system,” *PLoS One*, vol. 5, no. 4, p. e9862, 2010, doi: 10.1371/journal.pone.0009862.

[27] E. Gasteiger *et al.*, “Protein Identification and Analysis Tools on the ExPASy Server,” *The Proteomics Protocols Handbook*, pp. 571–607, 2005, doi: 10.1385/1-59259-890-0:571.

[28] D. Kozakov *et al.*, “The ClusPro web server for protein–protein docking,” *Nat. Protoc.*, vol. 12, no. 2, pp. 255–278, 2017, doi: 10.1038/nprot.2016.169.

[29] W. L. DeLano, “The PyMOL Molecular Graphics System,” *DeLano Scientific*, 2002.

[30] M. J. Abraham *et al.*, “GROMACS: High performance molecular simulations through multi-level parallelism from laptops to supercomputers,” *SoftwareX*, vol. 1–2, pp. 19–25, 2015, doi: 10.1016/j.softx.2015.06.001.

[31] D. W. A. Buchan and D. T. Jones, “The PSIPRED Protein Analysis Workbench: 20 years on,” *Nucleic Acids Res.*, vol. 47, no. W1, pp. W402–W407, 2019, doi: 10.1093/nar/gkz297.
